# Single-dose HPV vaccination in the United States — a multi-modeling analysis

**DOI:** 10.1016/j.lana.2025.101361

**Published:** 2026-01-10

**Authors:** Emily A. Burger, Jean-François Laprise, Jennifer C. Spencer, Stephen Sy, Mary Caroline Regan, Melanie Drolet, Éléonore Chamberland, Marc Brisson, Jane J. Kim

**Affiliations:** a Department of Health Policy and Management, Center for Health Decision Science, Harvard T.H. Chan School of Public Health, Boston, MA, USA; b Department of Health Management and Health Economics, University of Oslo, Oslo, Norway; c Centre de Recherche du CHU de Québec - Université Laval, Québec, Canada; d Department of Population Health, Department of Internal Medicine, University of Texas at Austin, Dell Medical School, Austin, TX, USA; e Département de Médecine Sociale et Préventive, Université Laval, Québec, Canada

**Keywords:** Cervical cancer, Human papillomavirus (HPV), Vaccines, Health policy, NIS-Teen, NHIS, Mathematical modeling

## Abstract

**Background:**

Evidence supporting the non-inferior efficacy of single-dose human papillomavirus (HPV) vaccination has prompted reconsideration of existing multi-dose HPV vaccination schedules. We evaluated the long-term health impact of adopting single-dose HPV vaccination in the United States to inform policy deliberations.

**Methods:**

We applied two validated individual-based simulation models of HPV transmission and cervical cancer to project the impact of switching from a two-dose to a single-dose HPV vaccination schedule in 2025 in the context of historical HPV vaccination uptake in the United States. Four scenarios were simulated: continuation of two-dose vaccination (or equivalent single-dose efficacy of 98%) and three alternative pessimistic single-dose strategies with lower vaccine efficacy (90%) and/or duration of protection (average of 25 years). Outcomes included age-standardized incidence rates of HPV-16 infection and cervical cancer from years 2005–2099. Additional analyses examined effects under lower vaccination coverage observed in select U.S. regions.

**Findings:**

Maintaining two doses or switching to a non-inferior single-dose HPV vaccination schedule was projected to nearly eliminate HPV-16 infections and reduce cervical cancer incidence by over 90% by the end of the century. Scenarios assuming a lower efficacy or waning protection showed increases in cervical cancer incidence of less than 2 percentage points decades after a switch to single-dose vaccination with no impact on the timeframe to cervical cancer elimination.

**Interpretation:**

Switching to a single-dose HPV vaccination schedule is projected to maintain reductions in cervical cancer, even under pessimistic efficacy and durability assumptions. Continued monitoring of single-dose HPV vaccine efficacy over time remains critical.

**Funding:**

PATH on behalf of the Single-Dose HPV Vaccine Evaluation Consortium; 10.13039/100000865Bill and Melinda Gates Foundation (grant No. OPP48979), and the 10.13039/100000002US National Institutes of Health/10.13039/100000054National Cancer Institute (Grant Number U01 CA253912).

**Research in context**

**Evidence before this study**

We conducted a search of PubMed on July 9, 2025, using the search terms “(single dose OR one dose) AND (human papillomavirus OR HPV) AND vaccine AND (modeling OR modelling)”. The search yielded 230 abstracts. After review, we identified several modeling studies that projected the long term health impact of single-dose human papillomavirus (HPV) vaccination, but few specifically examining scenarios of waning vaccine protection in the context of high-income countries. A study by Brisson and colleagues (2024) projected that switching from a two-dose to a one-dose program in high-income countries would not cause an appreciable rebound in HPV-16 infection, even under pessimistic waning assumptions (e.g., average protection of 25 years). The authors attributed this to high existing two-dose coverage and the timing of protection during peak sexual activity. Another study by Song and colleagues (2024) in the United Kingdom modeled immunity durations of 10 and 30 years, concluding that a single-dose schedule was highly cost-effective. In contrast, a modeling study by Daniels and colleagues (2022), also in the United Kingdom, projected that adopting a one dose regimen could result in a substantial number of additional HPV-related cancers over 100 years compared to a two-dose program, highlighting the uncertainty surrounding single-dose durability. While these studies provide crucial insights, projections of the health impact of single-dose HPV vaccination under waning protection scenarios remain limited for many specific high-income settings, including the United States with its unique demographic and vaccine coverage characteristics. Existing models have used different assumptions regarding the duration of protection, and their conflicting conclusions underscore the need for further analyses to inform policy decisions in countries considering a switch to a single-dose schedule, particularly in the United States where there were ongoing policy deliberations by the U.S. Centers for Disease Control and Prevention (CDC) Advisory Committee on Immunization Practices (ACIP).

**Added value of this study**

This is the first study to employ two independent mathematical models to evaluate the potential impact of a switch to single-dose HPV vaccination in the United States. By simulating various efficacy scenarios and accounting for potential waning protection, we provide a comprehensive analysis tailored to the unique demographic and coverage characteristics of the U.S. population. This modeling approach enhances the understanding of how a single-dose schedule might affect HPV-16 incidence and cervical cancer rates, ultimately informing policy deliberations. Our findings contribute to the existing literature by showcasing the potential public health impact of adopting a single-dose HPV vaccination strategy in the United States.

**Implications of all the available evidence**

The collective evidence from U.S. settings suggest that single-dose HPV vaccination may achieve similar reductions in HPV infections and cervical cancer incidence as the traditional two-dose regimen in settings like the United States. Even under pessimistic assumptions regarding vaccine efficacy and protection duration, our projections indicate that transitioning to a single-dose schedule could maintain substantial public health benefits. These findings underscore the importance of continued monitoring of long-term protection and efficacy to ensure the effectiveness of HPV vaccination strategies.

## Introduction

Cervical cancer, primarily caused by persistent infection with high-risk human papillomavirus (HPV), is preventable through prophylactic vaccination.^1^^,^^2^ In the United States (U.S.), HPV vaccination was introduced in 2006 as a three-dose schedule over six months. Emerging trial data led the U.S. Centers for Disease Control and Prevention (CDC) Advisory Committee on Immunization Practices (ACIP) to revise their HPV vaccine recommendations to two doses for individuals up to age 14 years. By 2022, accumulating empirical evidence supported the non-inferior efficacy of a single dose compared to two doses, prompting the World Health Organization (WHO) to endorse single-dose vaccine schedules for individuals up to age 20 years.^3^ By February 2025, high-income countries including the United Kingdom, Ireland, Australia, Spain, and Canada, had adopted single-dose HPV vaccination schedules.^4^ The ACIP was actively reconsidering its recommendations to potentially align with the shift towards single-dose HPV vaccination.

Recent findings from the ESCUDDO trial in Costa Rica^5^^,^^6^ confirm that single-dose HPV vaccination provides high protection against persistent infection with high-risk HPV types—comparable to that of multi-dose regimens—and reinforce earlier findings from the KEN SHE trial in Kenya^7^^,^^8^ and the DORIS trial in Tanzania.^9^ Although single-dose efficacy ranges from 92 to 99%, concerns remain regarding the durability of protection. Long-term studies, such as the Costa Rica Vaccine Trial (CVT) Long Term Follow-Up Study^10^ and the IARC Indian cohort study,^11^ support sustained antibody levels up to 16 years post-vaccination. While these follow-up times exceed those available during prior 2- or 3-dose policy decisions, data beyond 16 years are lacking. Two prior model-based analyses drew promising conclusions about single-dose HPV vaccination in high-income countries^12^^,^^13^; however, the applicability of these findings to the U.S. population, with its unique demographic and coverage characteristics, necessitates further exploration.

To address these uncertainties, mathematical simulation models can project the long-term impact of single-dose HPV vaccination, particularly under hypothetical waning scenarios. In this analysis, we use two independent mathematical models to evaluate a switch to single-dose HPV vaccination in the U.S. under various scenarios, to inform policy deliberations in the United States.

## Methods

### Analytic overview

We employed two independently-developed and validated simulation models of HPV transmission and cervical cancer—Harvard [Harvard T.H. Chan School of Public Health] and HPV-ADVISE [Université Laval]—that have been used in prior policy deliberations of the ACIP in the U.S. and for WHO's global cervical cancer elimination strategy.^14^, ^15^, ^16^, ^17^ Both models, adapted to the U.S. population, were used to simulate four single-dose HPV vaccination scenarios of varying efficacy and duration of protection.

**Fig. 1:**
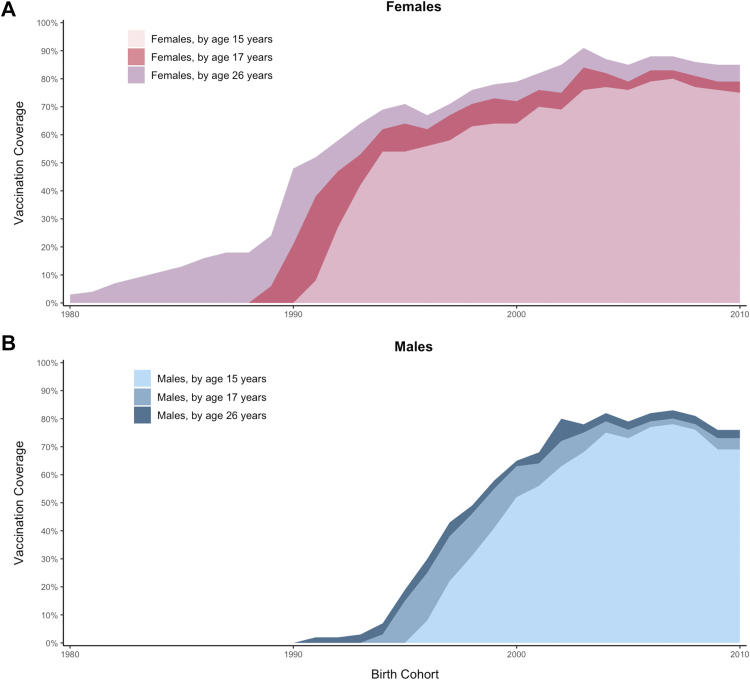
Model output of cumulative coverage of one or more doses of human papillomavirus (HPV) vaccination by birth cohort year for A) females and B) males by ages 15, 17 and 26 years based on primary data inputs from the National Immunization Survey (NIS)-Teen and National Health Interview Study (NHIS). See Methods.

For each scenario, the models projected the relative change in age-standardized HPV-16 incidence and cervical cancer incidence (attributable to all high-risk HPV genotypes) across multiple birth cohorts in the U.S. population from 2005 to 2099. HPV-16 is the genotype most difficult to control and would theoretically have the greatest rebound.^12^ We performed analyses in the context of the national average of U.S. HPV vaccination coverage rates following HPV vaccination introduction in 2006 (Fig. 1). In sensitivity analysis, we evaluated the four vaccination scenarios under lower vaccination coverage rates to reflect select U.S. states and counties with lower coverage and consequently lower herd immunity.

### Model descriptions and validation

**Fig. 2:**
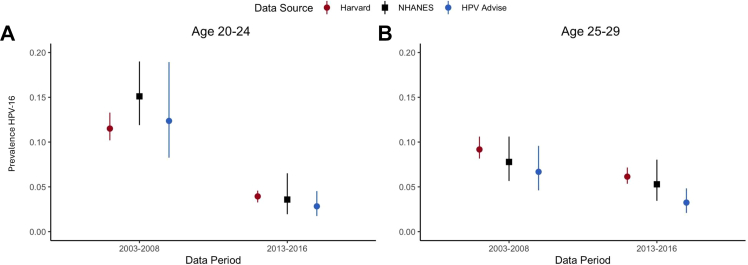
Model comparisons to human papillomavirus (HPV)-16 prevalence from the National Health and Nutrition Examination Survey (NHANES) pre- (2003–2008) and post- (2013–2016) HPV vaccine introduction for the Harvard and HPV-ADVISE models for A) ages 20−24, and B) ages 25−29 years. Both models reflect U.S. national HPV vaccination coverage rates based on National Immunization Survey (NIS-Teen) and National Health Interview Study (NHIS) (See methods). Model ranges capture the 10% and 90% credible intervals across good-fitting parameter sets.^18^^,^^19^ Ranges in data reflect the 95% confidence intervals.

The Harvard and HPV-ADVISE models, developed to simulate HPV transmission and cervical disease progression, have been described previously.^14^^,^^16^^,^^18^^,^^19^ Although they differ in their underlying structure and assumptions (Table 1), both models incorporate U.S. sexual behavior and mixing patterns across varying risk groups and have been calibrated (i.e., fit) to U.S. data on sexual mixing and pre-HPV vaccination epidemiology in the U.S.^18^^,^^19^ Both models integrated data on reported partnerships in the last year and show good correspondence with U.*S. data* in terms of the cumulative number of lifetime sexual partnerships (Supplement Figure S3). Both the HPV-ADVISE and Harvard models simulate the acquisition and progression of multiple HPV types independently. Contextualized to historical HPV vaccination coverage in the U.S. (Fig. 1), both models have also been validated against post-vaccine data in the U.S. (Fig. 2).^16^^,^^18^^,^^19^

**Table 1:** Key attributes of the Harvard and HPV-ADVISE simulation models.

Category	Harvard model	HPV-ADVISE model
Model type	Individual-based sexual transmission model (hybrid model using two, linked individual-based simulation models)	Individual-based sexual transmission model
Population	Females and males; population-based (multi-cohort)	
HPV genotypes	Independent HPV-16, -18, -31, -33, -45, -52, -58 + pooled high-risk + pooled low-risk	Independent HPV-16, -18, -6, -11, -31, -33, -45, -52, -58, -35, -39, -51, -56, -59, -66, -68, -73, and -82
Type of mixing and risk groups	Heterosexual mixing among 4 levels of sexual activity and single-year age	Heterosexual mixing among 4 levels of sexual activity and 12 age groups
Captures herd immunity	Yes	
HPV transmission	Probability per month of partnership duration (sex and HPV genotype-specific)	Probability per sexual act (sex and HPV genotype-specific)
Acquisition of HPV infection following exposure	Dependant on probability of transmission of the genotype and the individual’s history of prior infection to the genotype, in absence of vaccination (e.g., type-specific lifelong natural immunity)	
Health states	Tracks HPV progression through cervical cancers natural history health states (No HPV, HPV, CIN2, CIN3, cervical cancer, death)	Tracks HPV progression through cervical cancer natural history health states (No HPV, HPV, CIN1, CIN2, CIN3, cervical cancer, death)
Clearance and progression of natural history health states	Dependent on duration in health state and HPV genotype, in the absense of screening	Dependent on health state and HPV genotype-specific rates of progression and regression/clearance, in the absence of screening
Screening modeled for the single-dose analysis	Cytology-based screening (adherence consistent with previous analysis^14^)	Cytology-based screening (adherence consistent with previous analysis^16^^,^^18^)
Vaccine efficacy mechanism modeled for the single-dose analysis	Degree/Leaky^a^	Take/All-or-nothing^b^
Vaccine waning mechanism modeled for the single-dose analysis	Average duration of protection (time from protected to completely unprotected in each vaccinated person) is normally distributed	

a Vaccine reduces the probability of HPV infection by a certain percentage across all vaccinated individuals, thereby allowing partial susceptibility to infection (for each exposure to the infection).

b Assumes that a certain proportion of vaccinated individuals gain full immunity, while the remaining proportion remains entirely susceptible.

Briefly, the Harvard modeling framework integrates two individual-based models: the “Harvard-HPV” model,^19^ which simulates heterosexual HPV transmission dynamics, and the “Harvard-CC” model,^20^ which simulates the natural history of HPV-induced squamous cell cervical carcinogenesis. Both Harvard models were adapted to reflect the U.S. context. The U.S. Harvard-HPV model projects the percentage change in HPV incidence by age, cohort and genotype for each vaccination scenario relative to a no-vaccination baseline. These projections serve as inputs for the U.S. Harvard-CC model, which can track multiple birth cohorts of women from age nine through monthly duration-, genotype- and health state-dependent transitions across cervical cancer-related health states until death. Model outputs for each scenario represent the best-fitting Harvard-HPV and Harvard-CC identified through calibration to U.S. data.

The HPV-ADVISE model is an individual-based, transmission-dynamic model designed to simulate HPV infection and disease progression,^18^ as well as HPV vaccination and screening. HPV-ADVISE simulates 18 HPV types separately, including all types covered by the nonavalent vaccine. The model replicates U.S.-specific demography, heterosexual behavior, and HPV transmission dynamics, utilizing four distinct sexual activity risk groups and age-specific mixing patterns. Model outputs represent the mean of projections from the 50 best-fitting parameter sets identified through calibration to U.S. data to capture uncertainty.

### Vaccination scenarios: single-dose efficacy and durability

*Scenario 1* reflected continued use of the two-dose HPV vaccination program, or equivalently, a single-dose program with comparable protection, providing 98% efficacy in both sexes against vaccine-targeted HPV types included in the nonavalent vaccine, based on results from the KEN SHE and ESCUDDO randomized trial.^5^^,^^7^^,^^8^ This scenario assumed lifelong protection against new infections. *Scenarios 2–4* modeled a national policy switch in 2025 to a single-dose schedule for females and males up to age 20 years (aligned with the WHO recommendation), under varying assumptions of efficacy and duration. *Scenario 2* assumed 98% efficacy with an average duration of 25 years. *Scenario 3* assumed 90% efficacy—the lower bound reported in early KEN SHE results^7^—with lifelong protection. *Scenario 4* combined the lower efficacy (90%) with an average protection duration of 25 years.

Vaccine efficacy was implemented using distinct mechanisms in the two models. The Harvard model assumes each person receives a “leaky” vaccine benefit resulting in a cumulative protection of 98% (vaccine “degree”) against vaccine-targeted HPV genotypes. The efficacy input is calibrated to match the observed efficacy after five years, consistent with the reporting timelines of the KEN SHE clinical trial. In contrast, the HPV-ADVISE model assumes that individuals either fully respond or fully do not respond (vaccine “take”); as such, vaccine efficacy of 98% (base case) assumes 98% of individuals are fully protected by vaccination while 2% receive no protection. In both models, the vaccine does not affect the natural history among individuals already infected with HPV prior to vaccination. Consequently, both models inherently account for reduced vaccine effectiveness at older ages, since individuals vaccinated at older ages are more likely to have already acquired HPV infection, and the vaccine provides only prophylactic protection.

Despite no evidence suggesting that single-dose protection against HPV infections diminishes over time, uncertainty remains about the duration of this protection beyond what has been observed in clinical studies. In *Scenarios* *2* *and 4*, both models assumed that individuals would experience protection for an average of 25 years, normally distributed with a standard deviation of 5 years. Specifically, each vaccinated individual in our models was assigned a unique duration of protection, sampled from this distribution. While previous modeling studies have examined different patterns of waning protection, we believe our approach aligns with observed empirical data and adopts a cautious perspective as demonstrated by Brisson and colleagues.^12^ It is further assumed that once this period of stable protection concludes, the level of protection would immediately disappear at the individual level. These assumptions imply that 1) ∼2% of the vaccinated population will have no protection after 15 years, 2) 50% will have no protection after 25 years, and 3) ∼2% will have protection longer than 35 years.

### Historical HPV vaccination coverage

U.S. historical HPV vaccination coverage was based on primary analysis of individual-level data from U.S. national surveys, reconstructing HPV vaccination coverage by year and birth-cohort by completed number of doses through age 26 years (Supplement Figures S1–S2). Cumulative HPV vaccination coverage for adolescent girls and boys between ages 9 and 17 years was based on provider-verified age at vaccination from multiple National Immunization Survey (NIS)-Teen surveys conducted between 2008 and 2022.^21^ For cumulative vaccination coverage among adults aged 18–26 years, we used self-reported adult vaccination uptake reported by the National Health Interview Study (NHIS) in all years where HPV vaccination was assessed (2008, annually from 2013 to 2018, and 2022).^22^ The age- and sex-specific annual probabilities of receiving 1 dose or ≥2 doses (for individuals not previously vaccinated) were calculated based on the observed changes in female and male coverage across the survey years (Supplement Tables S1–S4) enabling the models to accurately reflect cumulative coverage by age 15, 17 and 26 years (Fig. 1). For the proportion of individuals receiving only a single dose prior to 2025, we assumed the individuals would experience protection consistent with single-dose characteristics outlined in our scenarios. We captured the initial use of the quadrivalent vaccine in 2006 (directly protecting against HPV-16, -18, -6 and -11 infections), and the transition to the nonavalent vaccine (additionally targeting HPV-31, -33, -45, -52 and -58 infections) starting in 2015. For all scenario projections, we assumed individuals received the nonavalent vaccine at current age-specific vaccination coverage levels, i.e., age-specific probabilities of being vaccinated from survey year 2023 onwards were assumed to remain stable (see Supplement Appendix Part 1). In sensitivity analysis, we applied a factor of 0.6 to all historic and future vaccine probabilities, to yield a cumulative single-dose coverage of 65.2% and 55.3% for females and males by age 26 years, respectively, which corresponds to estimated coverage in selected geographic areas such as Mississippi, Kentucky, Texas–Hidalgo County, and Wyoming.^23^

### Cervical cancer screening

All scenarios assumed cytology-based screening practice patterns for women aged 21–65 years, consistent with U.S. guidelines since 2012, with follow-up management based on established algorithms.^24^ Screening adherence assumptions mirrored previous ACIP analyses conducted for both models.^14^^,^^16^^,^^18^

## Results

**Fig. 3:**
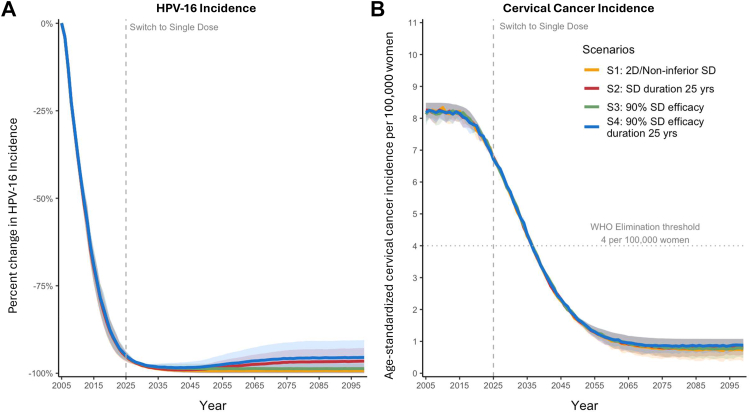
Projected reductions in age-standardized human papillomavirus (HPV)-16 incidence (**Panel A**) and age-standardized cervical cancer incidence (**Panel B**) from 2005–2099 across four two-dose (2D) and single-dose (SD) vaccination scenarios averaged across the Harvard and HPV-ADVISE models under national U.S. HPV vaccination coverage and a national triennial, cytology-based screening program. The WHO 2015 female population was used for standardization, consistent with WHO recommendations for cervical cancer elimination projections.^25^ Analysis outcomes were presented as the average of the Harvard and HPV-ADVISE models with uncertainty bounds showing the variability in the model projections, i.e., the minimum and maximum projections across the two models. Individual model results are presented in the Supplementary Material.

Under a scenario in which the United States continues its two-dose nonavalent HPV vaccination program–or transitions in 2025 to single-dose vaccination with equivalent protection—the Harvard and HPV-ADVISE models projected a mean reduction of 99.3% (minimum across models: 98.5%; maximum across models: 100%) in new HPV-16 infections (Fig. 3A) and a mean reduction of 91.3% (87.3%–95.4%) in cervical cancer incidence (Fig. 3B) by the end of the century, compared with a no-vaccination scenario. Even when assuming a lower bound of single-dose efficacy at 90%, the projected population-level impact remained similar to that of the two-dose regimen. In the pessimistic scenarios in which a single dose provided protection for an average of 25 years—whether paired with full or reduced efficacy—our models projected small increases in HPV-16 incidence beginning mid-century (∼2050). By the end of the century, the reduction in HPV-16 incidence was ∼4 (0.0–8.0) fewer percentage points (from 99.3% to 95.3%), and the reduction in cervical cancer incidence from any high-risk HPV genotypes was less than ∼2 (0.0–3.8) fewer percentage points (from 91.3% to 89.4%), compared to the base-case (non-inferior) single-dose scenario. Notably, none of the single-dose scenarios altered the projected timing for achieving the WHO's cervical cancer elimination threshold of fewer than four cases per 100,000 women. Individually, the Harvard and HPV-ADVISE models produced broadly consistent population-level impacts across the HPV vaccination scenarios; however, the Harvard model generally projected less variations across the vaccination scenarios compared with HPV-ADVISE (Supplement Figures S4–S7).

### Sensitivity analysis

**Fig. 4:**
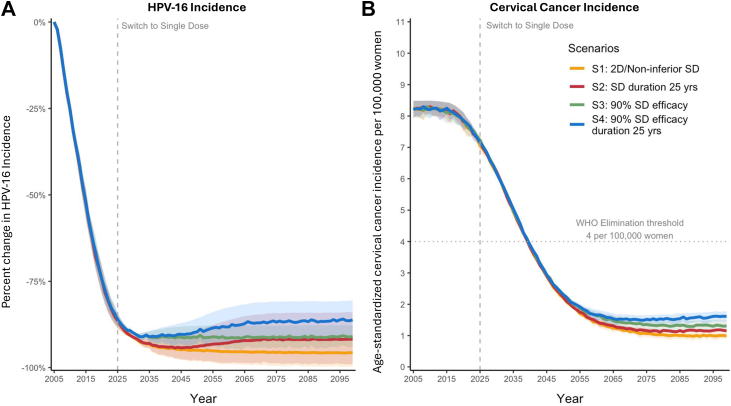
Projected reductions in age-standardized human papillomavirus (HPV)-16 incidence (**Panel A**) and age-standardized cervical cancer incidence (**Panel B**) from 2005–2099 across four two-dose (2D) and single-dose (SD) vaccination scenarios averaged across the Harvard and HPV-ADVISE models under lower HPV vaccination coverage and a national triennial, cytology-based screening program. The WHO 2015 female population was used for standardization, consistent with WHO recommendations for cervical cancer elimination projections.^25^ Analysis outcomes were presented as the average of the Harvard and HPV-ADVISE models with uncertainty bounds showing the variability in the model projections, i.e., the minimum and maximum projections across the two models. Individual model results are presented in the Supplementary Material.

In sensitivity analyses evaluating lower vaccination coverage (as observed in different geographic regions), our models projected reduced overall protection against HPV-16 infections and cervical cancer compared to projections based on higher national average coverage (Fig. 4; Supplement Figures S8–S11). The impact of the pessimistic scenarios assuming lower single-dose efficacy and waning protection on changes in HPV-16 incidence and cervical cancer remained relatively modest but were more pronounced in the lower-coverage setting compared with higher national coverage. For example, by the end of the century, reductions in HPV-16 infections and cervical cancer incidence were 9.9 (9.1%–10.8%) and 7.4 (6.9–7.9) percentage points lower, respectively, compared with the low coverage projections under a single-dose scenario with 98% efficacy and lifelong protection (versus ∼4 (0.0–8.0) and ∼2 (0.0–3.8) percentage points in the national average scenario).

## Discussion

Grounded in evidence from clinical studies, our multi-modeling analysis found that single-dose HPV vaccination has similar effectiveness against HPV-16 infections and cervical cancer as a two-dose regimen. Even under pessimistic assumptions—such as protection lasting only 25 years—the projected reductions in HPV infections and cervical cancer incidence compared with no vaccination were similar to those assuming high, lifelong protection. This insensitivity to duration of protection out to 25 years reflects both the few number of new sexual partnerships after protection wanes and herd effects established through high population-level coverage. In lower-coverage regions, projected declines in HPV-16 infections and cervical cancer incidence remained substantial, with only modest attenuation—by 9.9 and 7.4 percentage points, respectively, under worst-case scenario assumptions.

A strength of this analysis is the use of two independently-developed models adapted to the U.S. context. Differences in projected rebounds of HPV-16 infections were primarily driven by two factors: (1) the distribution of cumulative sexual partnerships by age, and (2) the vaccine efficacy mechanism. In HPV-ADVISE, more lifetime partnerships occurred after age 30 years than in the Harvard model, resulting in higher exposure after the vaccine wanes. The Harvard model's “degree” efficacy mechanism reduces infection risk without conferring complete immunity, leading to greater sensitivity to the lower vaccine coverage and efficacy assumptions than HPV-ADVISE. HPV-ADVISE applies a “take” mechanism, where a fixed proportion of individuals are fully protected. Importantly, both models closely matched observed HPV-16 prevalence reported in NHANES before (2003–2008) and after (2013–2016) HPV vaccine introduction. Although NHANES data were not available for more recent years, our model projections align well with findings from a recent epidemiological study reporting HPV prevalence among adolescent girls and young women in 2021–2023.^26^ The observed difference in projected cervical cancer incidence between the models is largely attributable to the proportion of squamous cell carcinomas assigned to vaccine-targeted HPV genotypes, which is lower in the Harvard model than in HPV-ADVISE. These ranges reflect the results of model calibration and produce model outputs that reflect data uncertainties. Despite structural differences, both models reach similar conclusions.

Our study also reconstructed detailed U.S. historical vaccination coverage using nationally representative surveys capturing evolving patterns across birth cohorts, including individuals receiving only one dose—who are often underrepresented in other analyses. This broader uptake contributes to more rapid declines in HPV and cervical cancer.

Our findings align with prior high-income country analyses,^12^^,^^13^^,^^27^ though our projections—particularly under pessimistic waning assumptions—suggest a smaller rebound compared with the two Canadian-based analyses. Notably, a prior analysis using the HPV-ADVISE model to broadly explore key factors that impact single-dose vaccine effectiveness in high-income countries, projected a higher rebound assuming single-dose waning,^12^ largely due to differences in vaccination coverage assumptions and sexual activity. The previous analysis assumed school-based vaccination programs with high coverage by age 10 years, whereas our analysis reflects broader uptake across ages 9–26 years (average 15 years) to specifically reproduce vaccine coverage in the U.S. Consequently, in the current study, under the pessimistic scenario of 25 years of protection, individuals lose their protection at about age 40 years where they have few new sexual partners and thus a decreased chance of acquiring HPV (versus age 35 years in the previous analysis). Additionally, the previous analysis with HPV-ADVISE reflected data from Canada and other high-income countries, which had a higher average number of lifetime partners especially in older ages, resulting in a greater bounce back in HPV due to greater HPV exposure when the single-dose vaccine was assumed to wane. Notably, a study by Song et al.,^27^ a non-industry funded single-dose modeling study set in the United Kingdom, projected that even under a scenario of 80% efficacy and waning protection after 10 years, switching to a single-dose schedule would result in only about 1% additional cervical cancer cases compared to a two-dose program—suggesting an even more optimistic outlook than that reflected in our U.S.-based models.

This study has several limitations. We did not evaluate differential efficacy of single-dose vaccination in males, include non-cervical HPV-related cancers, or mitigation strategies—scenarios explored in prior work. For example, even if single-dose efficacy were reduced to 70% in males only, population-level protection would likely remain robust due to herd effects—assuming durable female immunity and high gender-neutral vaccination.^12^ Non-cervical cancers typically have a longer interval between infection and cancer onset, implying that even under pessimistic scenarios, any rebound in cancer burden would be delayed and attenuated. Moreover, should waning protection be observed in the future, modeling indicates that public health strategies—such as transitioning back to a two-dose regimen—could effectively mitigate adverse outcomes without requiring re-vaccination of individuals who initially received one dose.^13^^,^^28^

We assumed all detected HPV infections represent new acquisitions rather than potential reactivation of latent infections, which may impact the effectiveness of vaccination at older ages. Both models also overestimate number of new partners at older ages, which would bias our projections toward greater rebounds when assuming single-dose waning. We modeled only heterosexual transmission; while this may underestimate network complexity, high gender-neutral coverage provides indirect protection across sexual orientations. Nonetheless, inclusion of same-sex transmission dynamics could improve future projections. In addition, although some simulations showed vaccine-type HPV elimination at certain coverage levels, these reflect simplistic conditions that do not account for important heterogeneities in coverage and risk factors across regions or subpopulations, nor importation of infection through migration. While we have not captured the complex interplay between social determinants of health, healthcare access, screening, and HPV vaccine uptake in our analysis, we note that these factors may contribute to less robust population-level herd immunity in certain groups. Finally, we conservatively assumed triennial cytology-based screening. We deliberately simulated single-dose scenarios under cytology-based screening—which is less sensitive than HPV-based screening—to capture the greatest potential rebounds in cervical cancer incidence that may arise from waning vaccine protection. However, as primary HPV testing is also widely recommended, actual reductions in cervical cancer may exceed those projected, particularly in low vaccine efficacy or waning scenarios.

In the United States, where more nearly 45% of children are covered by publicly funded insurance programs such as CHIP or Medicaid, streamlining HPV vaccination to a single-dose schedule could allow resources currently dedicated to administering additional doses to be reallocated to other preventive health services and outreach efforts, thereby maximizing the overall public health benefit. In conclusion, given the robust uptake of HPV vaccination over multiple age groups, females and males, and over many years, single-dose HPV vaccination is projected to yield similar reductions in HPV infections and cervical cancer as two doses. Even under a pessimistic assumption of 25 years of protection, a transition to single-dose vaccination is projected to lead to a limited rebound in cervical cancer incidence in the U.S. Continued monitoring of long-term protection in clinical studies is essential for detecting any waning and guiding timely mitigation strategies, if needed.

## Contributors

Conceptualisation: EAB, JFL, MB, JJK; Formal analysis: EAB, JS, JFL, EC, MD; Funding acquisition: MB and JJK; Software: EAB, SS, JS, and JFL, and MB; Supervision: MB and JJK.

Validation/data verification: EAB, SS, JS, EC, JFL; Raw data access: EAB, SS, JS; Visualisation: MCR, JS, EAB; writing—original draft: EAB; writing—review & editing: All authors; Final approval for submission: All authors.

## Data sharing statement

Model outputs underpinning the analysis are available upon reasonable request.

## Declaration of interests

The authors have no conflicts to disclose.

## Appendix A Supplementary data

Supplementary Appendix

HPV-FRAME checklist
