# Supplementary material for: Single-dose HPV vaccination in the United States — a multi-modeling analysis
Source: Lancet Reg Health Am. 2026 Jan 10;55:101361. doi: 10.1016/j.lana.2025.101361 (PMC12825051; doi:10.1016/j.lana.2025.101361)
Supplement: Supplementary Appendix [file mmc1.pdf]

Supplementary Appendix to accompany the manuscript:
Single-Dose HPV Vaccination in the United States — A Multi-modeling Analysis

Contents:

1. **Additional model assumptions**
2. **Model fits**
3. **Additional results**
4. **Supplement Figures**
   a. Supplement Figure 1. Birth cohorts mapped to NIS-TEEN and NHIS survey years
   b. Supplement Figure 2. Completion of doses of human papillomavirus (HPV) vaccination by birth cohort for A) females one-dose or more B) females two-dose or more, and C) males one-dose or more and D) males two-dose or more for individuals aged 9–26 years using primary data from the National Immunization Survey (NIS)-Teen and National Health Interview Study (NHIS)
   c. Supplement Figure 3. Model output median cumulative lifetime number of partners for the A) Harvard and C) HPV-ADVISE models and the number of new partners for the B) Harvard and D) HPV-ADVISE models compared with real-world data from the U.S. (NHANES and NSFG), Canada (CONNECT), and United Kingdom (NATSAL-3).
   d. Supplement Figure 4. Projected reductions in age-standardized human papillomavirus (HPV)-16 incidence (Panel A) and age-standardized cervical cancer incidence (Panel B) from 2005–2099 shown separately for the Harvard and HPV-ADVISE models under national U.S. HPV vaccination coverage for *Scenario 1* (two-dose HPV vaccination or switch to single-dose vaccination in 2025 with equivalent protection)
   e. Supplement Figure 5. Projected reductions in age-standardized human papillomavirus (HPV)-16 incidence (Panel A) and age-standardized cervical cancer incidence (Panel B) from 2005–2099 shown separately for the Harvard and HPV-ADVISE models under national U.S. HPV vaccination coverage for *Scenario 2* (switch to single-dose vaccination in 2025 assuming an average duration of 25 years (5-year standard deviation))
   f. Supplement Figure 6. Projected reductions in age-standardized human papillomavirus (HPV)-16 incidence (Panel A) and age-standardized cervical cancer incidence (Panel B) from 2005–2099 shown separately for the Harvard and HPV-ADVISE models under national U.S. HPV vaccination coverage for *Scenario 3* (switch to single-dose vaccination in 2025 assuming single-dose vaccine efficacy of 90%)
   g. Supplement Figure 7. Projected reductions in age-standardized human papillomavirus (HPV)-16 incidence (Panel A) and age-standardized cervical cancer incidence (Panel B) from 2005–2099 shown separately for the Harvard and HPV-ADVISE models under national U.S. HPV vaccination coverage for *Scenario 4* (switch to single-dose vaccination in 2025 assuming single-dose vaccine efficacy of 90% and an average duration of 25 years (5-year standard deviation))
   h. Supplement Figure 8. Projected reductions in age-standardized human papillomavirus (HPV)-16 incidence (Panel A) and age-standardized cervical cancer incidence (Panel B) from 2005–2099 shown separately for the Harvard and HPV-ADVISE models under lower HPV vaccination coverage for *Scenario 1* (two-dose HPV vaccination or switch to single-dose vaccination in 2025 with equivalent protection)
   i. Supplement Figure 9. Projected reductions in age-standardized human papillomavirus (HPV)-16 incidence (Panel A) and age-standardized cervical cancer incidence (Panel B) from 2005–2099 shown separately for the Harvard and HPV-ADVISE models under lower HPV vaccination coverage for *Scenario 2* (switch to single-dose vaccination in 2025 assuming an average duration of 25 years (5-year standard deviation))
   j. Supplement Figure 10. Projected reductions in age-standardized human papillomavirus (HPV)-16 incidence (Panel A) and age-standardized cervical cancer incidence (Panel B) from 2005–2099 shown separately for the Harvard and HPV-ADVISE models under lower HPV vaccination coverage for *Scenario 3* (switch to single-dose vaccination in 2025 assuming single-dose vaccine efficacy of 90%)
   k. Supplement Figure 11. Projected reductions in age-standardized human papillomavirus (HPV)-16 incidence (Panel A) and age-standardized cervical cancer incidence (Panel B) from 2005–2099 shown separately for the Harvard and HPV-ADVISE models under lower HPV vaccination coverage for *Scenario 4* (switch to single-dose vaccination in 2025 assuming single-dose vaccine efficacy of 90% and an average duration of 25 years (5-year standard deviation))
5. **Supplement Tables**

a. Supplement Table 1. Model input for the annual probability of receiving 2+ doses of the HPV vaccine for females (shaded area reflects values projected for years 2023+)
b. Supplement Table 2. Model input for the annual probability of receiving 1 dose of the HPV vaccine for females (shaded area reflects values projected for years 2023+)
c. Supplement Table 3. Model input for the annual probability of receiving 2+ doses of the HPV vaccine for males (shaded area reflects values projected for years 2023+)
d. Supplement Table 4. Model input for the annual probability of receiving 1 dose of the HPV vaccine for males (shaded area reflects values projected for years 2023+)
e. Supplement Table 5. Model output of cumulative coverage of the human papillomavirus (HPV) vaccine by birth cohort, number of doses, year, sex and age (shaded area reflects accumulated coverage for future birth cohorts)
6. Supplement References

1. Additional analysis assumptions

Historical HPV vaccination coverage in the United States

The age- and sex-specific annual probabilities of being vaccinated (for individuals not already vaccinated) was based on the changes in female and male adolescent (provider-verified coverage reported in National Immunization Survey (NIS)-Teen interviews undertaken annually between 2008 and 2022 (see equation below). We reconstructed vaccination completion across birth cohorts by using provider-reported data on vaccination receipt, number of doses, and age at first dose by sex at the oldest available age (i.e., among those aged 17 years at the time of survey, except for 2022 where cumulative vaccination by age at survey was used for each included birth cohort). Data reflected an average of 3,500 adolescents per survey year who were aged 17 years at the time of the survey and had provider-verified vaccination data. Analyses applied provider-specific survey weights from NIS-Teen to reflect the US population, accounting for non-response.

For female and male vaccination completion coverage between ages 18–26 years-old, we relied on National Health Interview Study (NHIS) (self-reported). HPV vaccination, including age at receipt of first vaccine dose and number of doses was assessed in 2008, 2013 to 2018, and 2022. Age at HPV vaccine receipt was not assessed in 2008, therefore we assumed all reported vaccination had occurred in the prior year (2007). The number of HPV vaccine doses was not assessed in 2022, we assumed all individuals completed two doses. NHIS data reflected an average of 8,000 respondents per year from birth cohorts potentially eligible for HPV vaccine (born 1980 or later). Sample size was too small to estimate both cohort- and year-specific vaccination. Therefore, we first estimated catch-up vaccination for each birth cohort using self-report of vaccination between 18 and 26 years of age. We then calculated the proportion of catch-up vaccines received at each age by sex and distributed birth-cohort specific coverage, accordingly, producing table birth-cohort estimates. Analyses applied NHIS survey weights by year to reflect the U.S. population, accounting for non-response.

We mapped ages and survey years (**Supplement Figure 1**) to estimate cumulative birth cohort-specific vaccination coverage by number of completed doses combining data across the NIS-Teen and NHIS surveys (**Supplement Figure 2**), which were consistent with cumulative coverage achieved in the models (**Main Manuscript Figure 1**). Two-dose recipients were retrospectively classified as "up-to-date" in line with the Centers for Disease Control and Prevention (CDC) Advisory Committee on Immunization Practices' current recommendations. To calculate the probability that an unvaccinated individual becomes vaccinated between two time points (e.g., from age 13 in 2015 to age 14 in 2016), we used the following formula:

$$P_{vu} = \frac{V_2 - V_1}{1 - V_1}$$

Where:

- P_{vu} is the probability that an unvaccinated individual becomes vaccinated between the two time points.
- V_1 is the initial proportion of the cohort that is vaccinated at time 1.
- V_2 is the proportion of the cohort that is vaccinated at time 2.

The model inputs for the annual probability of receiving an HPV vaccine by age are available for females (**Supplement Tables 1 and 2**) and males (**Supplement Tables 3 and 4**) by number of doses completed, and for the model-implied accumulated vaccination coverage by birth cohort by age 17 and 26 years (**Supplement Table 5**). Age-specific probabilities of being vaccinated from survey year 2023 onwards were assumed to remain stable. We observed some inconsistent age-specific trends among females in 2020 and 2021 that were not maintained in 2022 and 2023 survey years, leading to temporary increases in coverage for several birth cohorts. Consequently, we adjusted annual probability inputs for females to ensure cumulative coverage with >=1 dose did not exceed 85% by age 26 years. Switching to single-dose scenarios were assumed to start in 2025.

HPV-16 prevalence comparisons

For model comparisons (**Main Manuscript Figure 2**), we analyzed HPV-16 prevalence in the period prior to the introduction of HPV vaccination (2003–2008) and after HPV (2013–2016) vaccine introduction, in the years available by prevalence from the National Health and Nutrition Examination Survey (NHANES). NHANES is completed in

two-year cycles; for pre-vaccination estimates we combined cycles 2003-2004, 2005-2006, and 2007-2008. For post-vaccination estimates we combined data from 2013-2014 and 2015-2016, as data from 2017-2018 are not publicly available and data from 2019-2020 and 2021-2022 do not include HPV prevalence due to the COVID-19 pandemic. We combined laboratory data on HPV prevalence from linear array, assessed from self-collected vaginal swabs as part of the NHANES physical exam, including a total of 5,136 women in the 2003-2008 combined sample and 3,867 women in the 2013-2016 combined sample (1). HPV prevalence was reported for those 20-24 years and 25-29 years, reflecting the age groups for whom vaccine impact would be highest. Analyses incorporated cycle-specific survey weights to reflect the US population, accounting for non-response. Comparisons were made to NHANES data using model year 2015 in both models.

Modeled scenarios

A summary of modeled scenarios is provided in **Supplement Table 6**.

2. Model fits

Cumulative lifetime number of partners

Overall, the NHANES and NHIS survey conducted in the United States report a lower cumulative lifetime of number of partners compared with Canada and the United Kingdom (**Supplement Figure 3**), settings that have been the focus of prior single-dose HPV vaccination analyses (2-4). Both Harvard (**Supplement Figure 3A)** and HPV-ADVISE (**Supplement Figure 3B**) show good correspondence with data from the U.S.; however, both models project a greater number of new partnerships in older ages compared with the data, partially explained by age, period and cohort effects in the data.

3. Additional results

Additional analysis results for each of the four HPV vaccination scenarios disaggregated by the Harvard and HPV-ADVISE models are provided in **Supplement Figures 4-7** and for the lower vaccine coverage sensitivity analysis in **Supplement Figures 8-11**. The WHO 2015 female population was used for standardization, consistent with WHO recommendations for cervical cancer elimination projections (5).

4. Supplement Figures

Supplement Figure 1. Birth cohorts mapped to NIS-TEEN and NHIS survey years

Survey Year	Age 13	14	15	16	17	18	19	20	21	22	23	24	25	26
2008	1995	1994	1993	1992	1991	1990	1989	1988	1987	1986	1985	1984	1983	1982
2009	1996	1995	1994	1993	1992	1991	1990	1989	1988	1987	1986	1985	1984	1983
2010	1997	1996	1995	1994	1993	1992	1991	1990	1989	1988	1987	1986	1985	1984
2011	1998	1997	1996	1995	1994	1993	1992	1991	1990	1989	1988	1987	1986	1985
2012	1999	1998	1997	1996	1995	1994	1993	1992	1991	1990	1989	1988	1987	1986
2013	2000	1999	1998	1997	1996	1995	1994	1993	1992	1991	1990	1989	1988	1987
2014	2001	2000	1999	1998	1997	1996	1995	1994	1993	1992	1991	1990	1989	1988
2015	2002	2001	2000	1999	1998	1997	1996	1995	1994	1993	1992	1991	1990	1989
2016	2003	2002	2001	2000	1999	1998	1997	1996	1995	1994	1993	1992	1991	1990
2017	2004	2003	2002	2001	2000	1999	1998	1997	1996	1995	1994	1993	1992	1991
2018	2005	2004	2003	2002	2001	2000	1999	1998	1997	1996	1995	1994	1993	1992
2019	2006	2005	2004	2003	2002	2001	2000	1999	1998	1997	1996	1995	1994	1993
2020	2007	2006	2005	2004	2003	2002	2001	2000	1999	1998	1997	1996	1995	1994
2021	2008	2007	2006	2005	2004	2003	2002	2001	2000	1999	1998	1997	1996	1995
2022	2009	2008	2007	2006	2005	2004	2003	2002	2001	2000	1999	1998	1997	1996

Supplement Figure 2. Completion of doses of human papillomavirus (HPV) vaccination by birth cohort for A) females one-dose or more B) females two-dose or more, and C) males one-dose or more and D) males two-dose or more for individuals aged 9–26 years using primary data from the National Immunization Survey (NIS)-Teen and National Health Interview Study (NHIS)

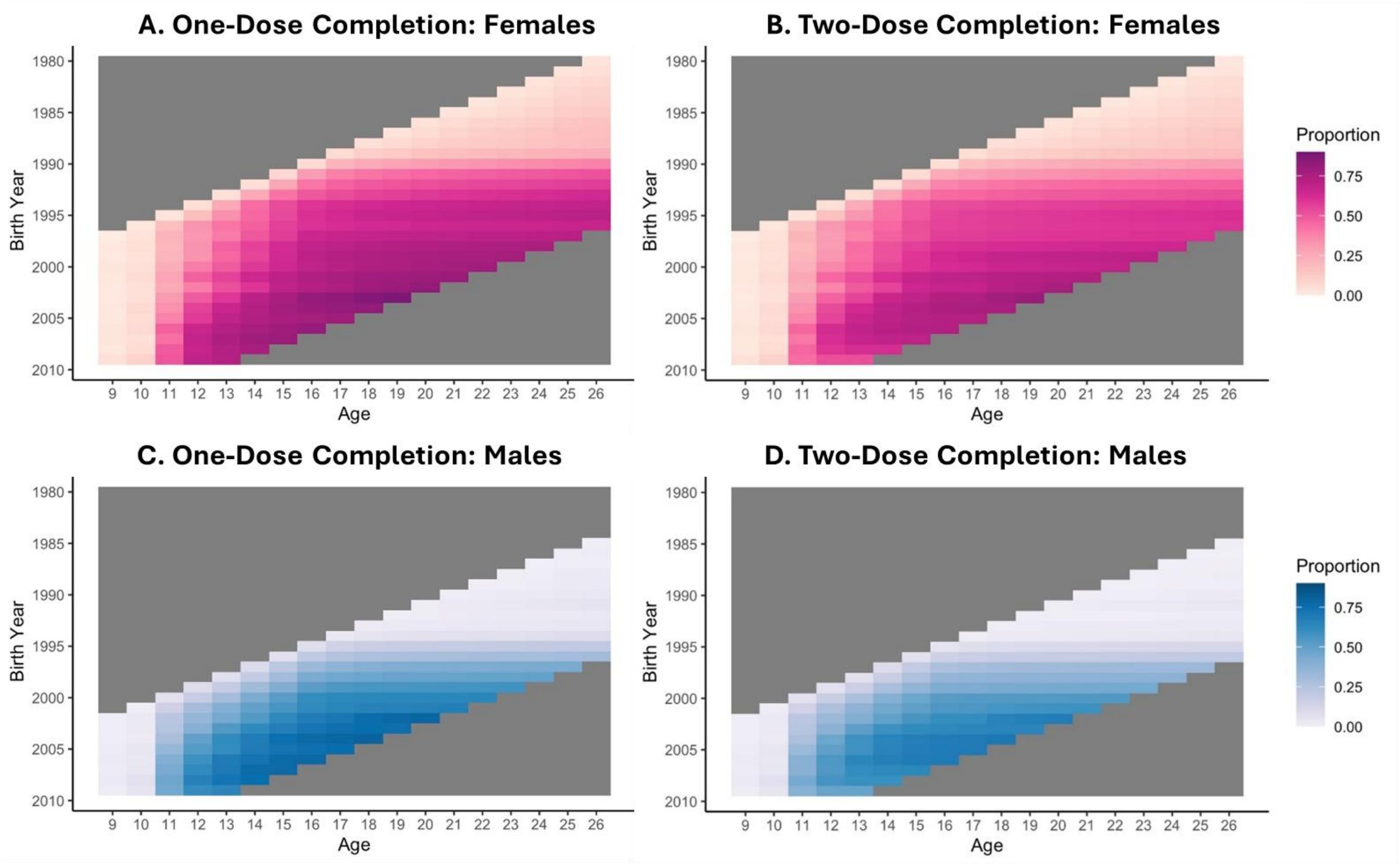

Supplement Figure 3. Model output median cumulative lifetime number of partners for the A) Harvard and C) HPV-ADVISE models and the number of new partners for the B) Harvard and D) HPV-ADVISE models compared with real-world data from the U.S. (NHANES and NSFG), Canada (CONNECT), and United Kingdom (NATSAL-3). Decreasing cumulative number of lifetime partnerships in the real-world data are explained by age, period and cohort effects. New partners is calculated as a difference from the next-youngest age group using 5-year buckets in both the models and data. Harvard model ranges reflect the 25th and 75th percentile of individuals within the model. HPV-ADVISE model ranges reflect the 10th and 90th percentiles of the good-fitting parameter sets.

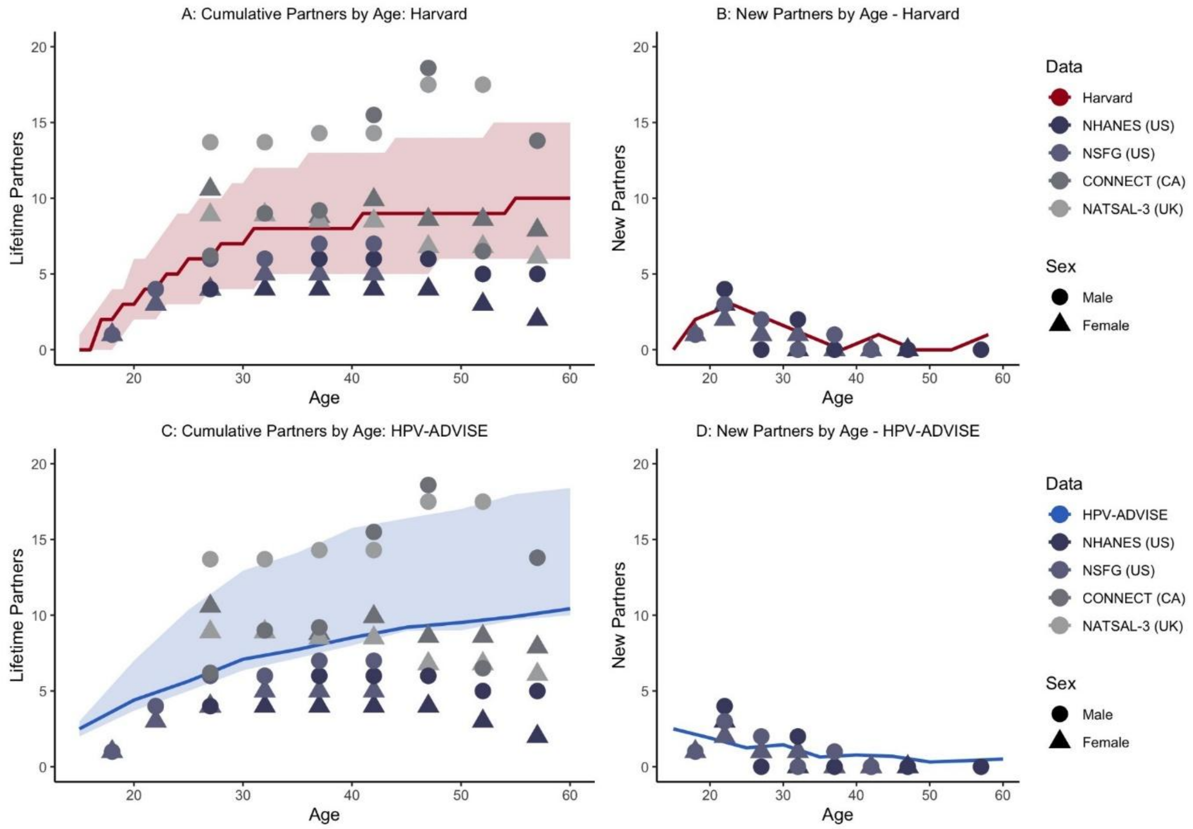

Supplement Figure 4. Projected reductions in age-standardized human papillomavirus (HPV)-16 incidence (**Panel A**) and age-standardized cervical cancer incidence (**Panel B**) from 2005–2099 shown separately for the Harvard and HPV-ADVISE models under national U.S. HPV vaccination coverage for *Scenario 1* (two-dose HPV vaccination or switch to single-dose vaccination in 2025 with equivalent protection). The WHO 2015 female population was used for standardization, consistent with WHO recommendations for cervical cancer elimination projections (5).

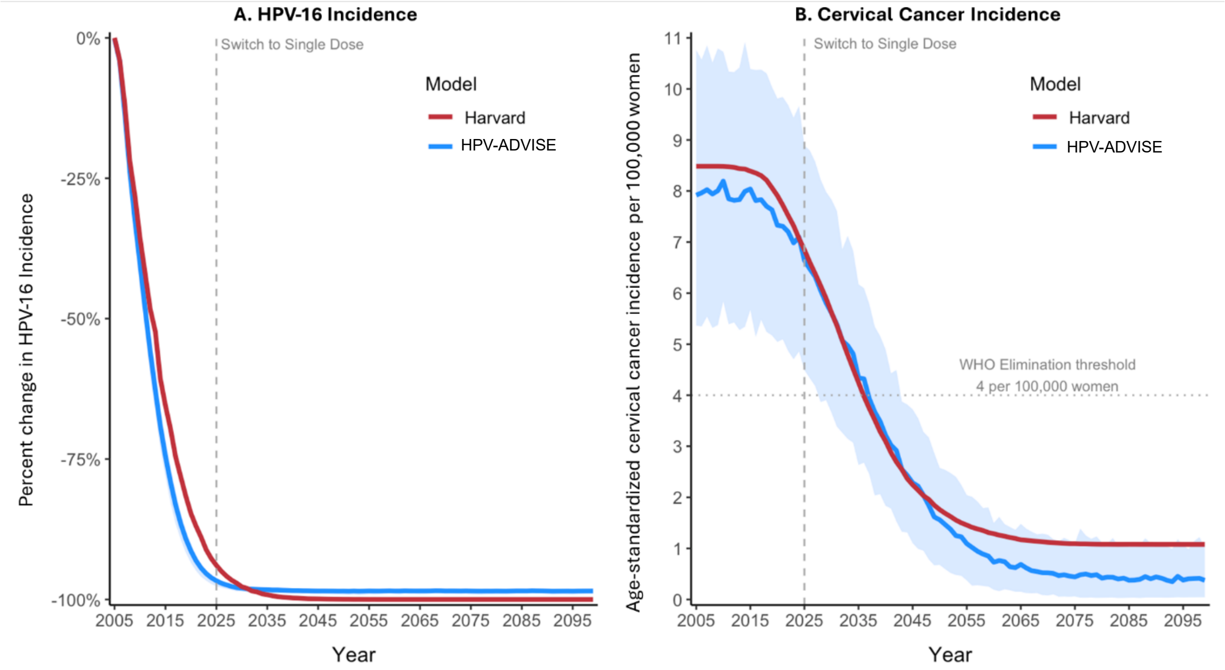

Supplement Figure 5. Projected reductions in age-standardized human papillomavirus (HPV)-16 incidence (**Panel A**) and age-standardized cervical cancer incidence (**Panel B**) from 2005–2099 shown separately for the Harvard and HPV-ADVISE models under national U.S. HPV vaccination coverage for *Scenario 2* (switch to single-dose vaccination in 2025 assuming an average duration of 25 years (5-year standard deviation)). The WHO 2015 female population was used for standardization, consistent with WHO recommendations for cervical cancer elimination projections (5).

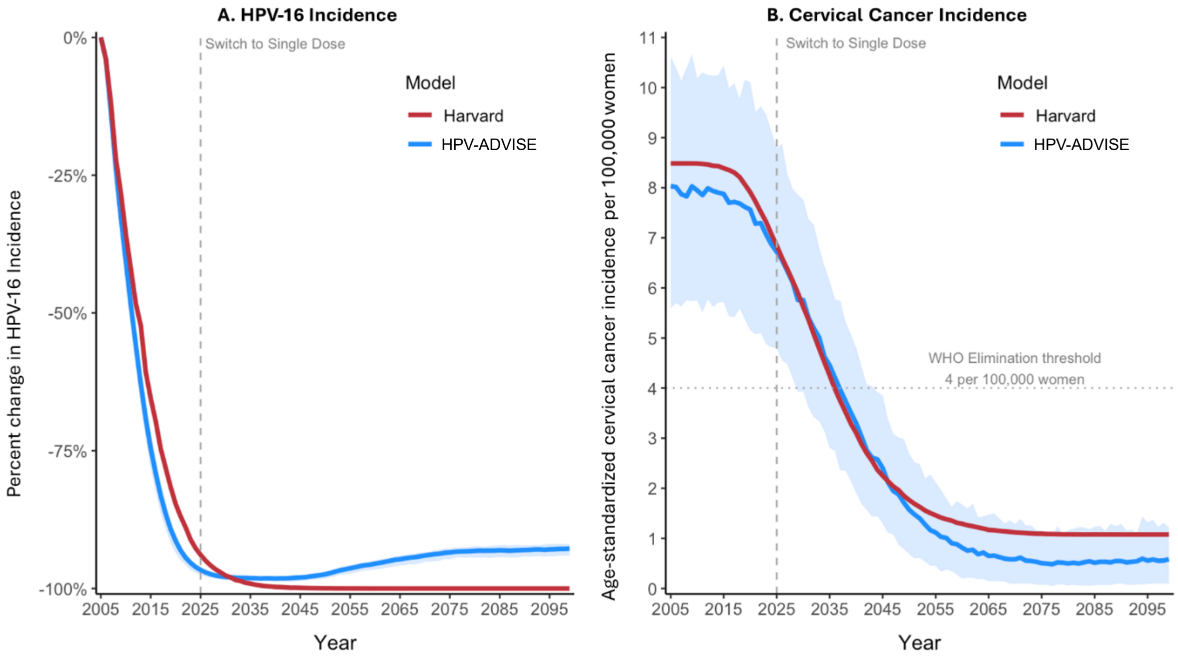

Supplement Figure 6. Projected reductions in age-standardized human papillomavirus (HPV)-16 incidence (**Panel A**) and age-standardized cervical cancer incidence (**Panel B**) from 2005–2099 shown separately for the Harvard and HPV-ADVISE models under national U.S. HPV vaccination coverage for *Scenario 3* (switch to single-dose vaccination in 2025 assuming single-dose vaccine efficacy of 90%). The WHO 2015 female population was used for standardization, consistent with WHO recommendations for cervical cancer elimination projections (5).

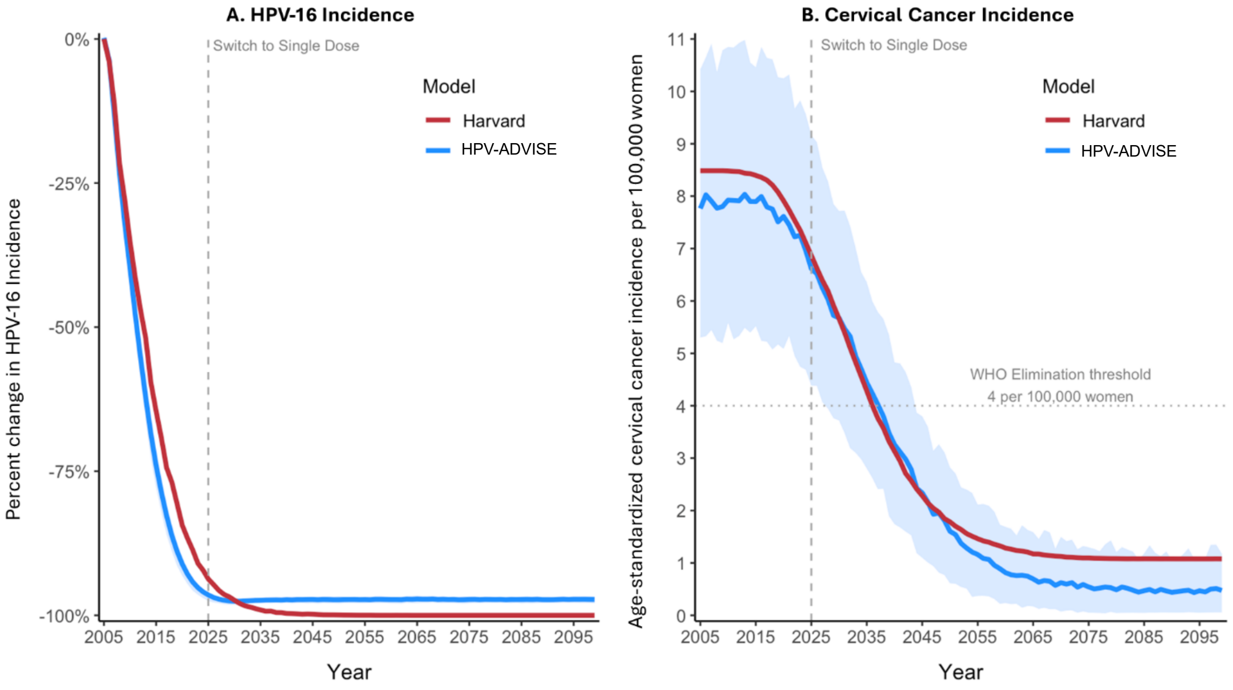

Supplement Figure 7. Projected reductions in age-standardized human papillomavirus (HPV)-16 incidence (**Panel A**) and age-standardized cervical cancer incidence (**Panel B**) from 2005–2099 shown separately for the Harvard and HPV-ADVISE models under national U.S. HPV vaccination coverage for *Scenario 4* (switch to single-dose vaccination in 2025 assuming single-dose vaccine efficacy of 90% and an average duration of 25 years (5-year standard deviation)). The WHO 2015 female population was used for standardization, consistent with WHO recommendations for cervical cancer elimination projections (5).

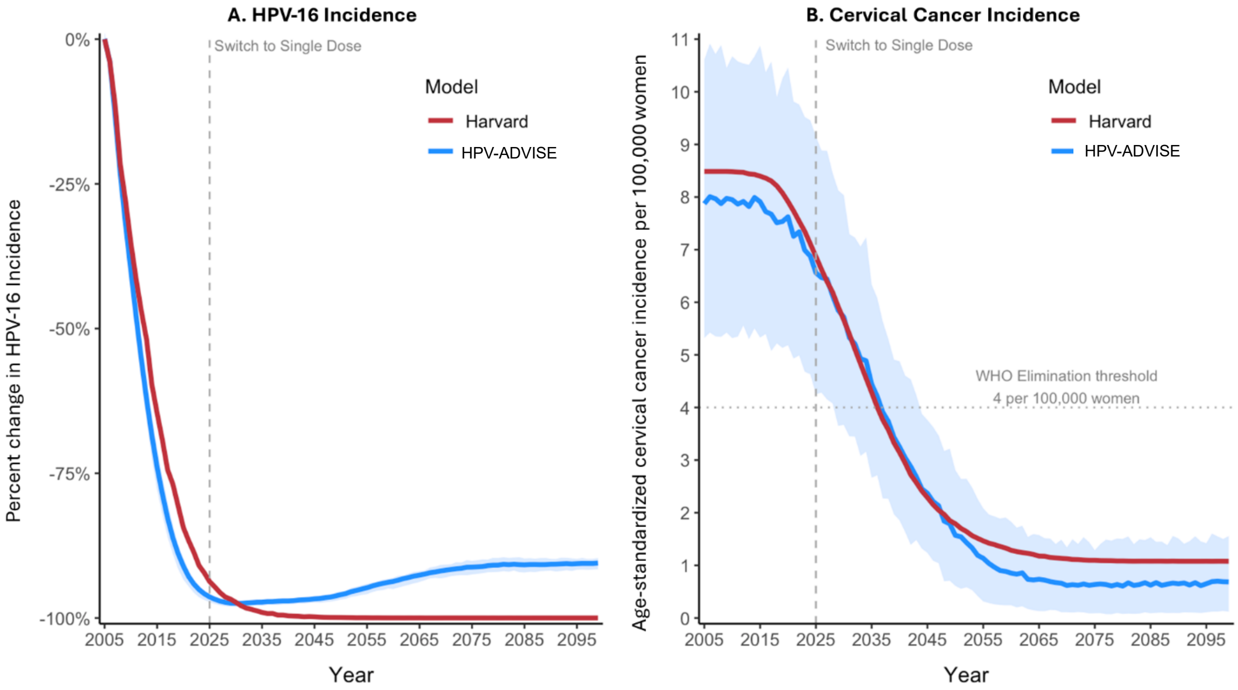

Supplement Figure 8. Projected reductions in age-standardized human papillomavirus (HPV)-16 incidence (**Panel A**) and age-standardized cervical cancer incidence (**Panel B**) from 2005–2099 shown separately for the Harvard and HPV-ADVISE models under <u>lower</u> HPV vaccination coverage for *Scenario 1* (two-dose HPV vaccination or switch to single-dose vaccination in 2025 with equivalent protection). The WHO 2015 female population was used for standardization, consistent with WHO recommendations for cervical cancer elimination projections (5).

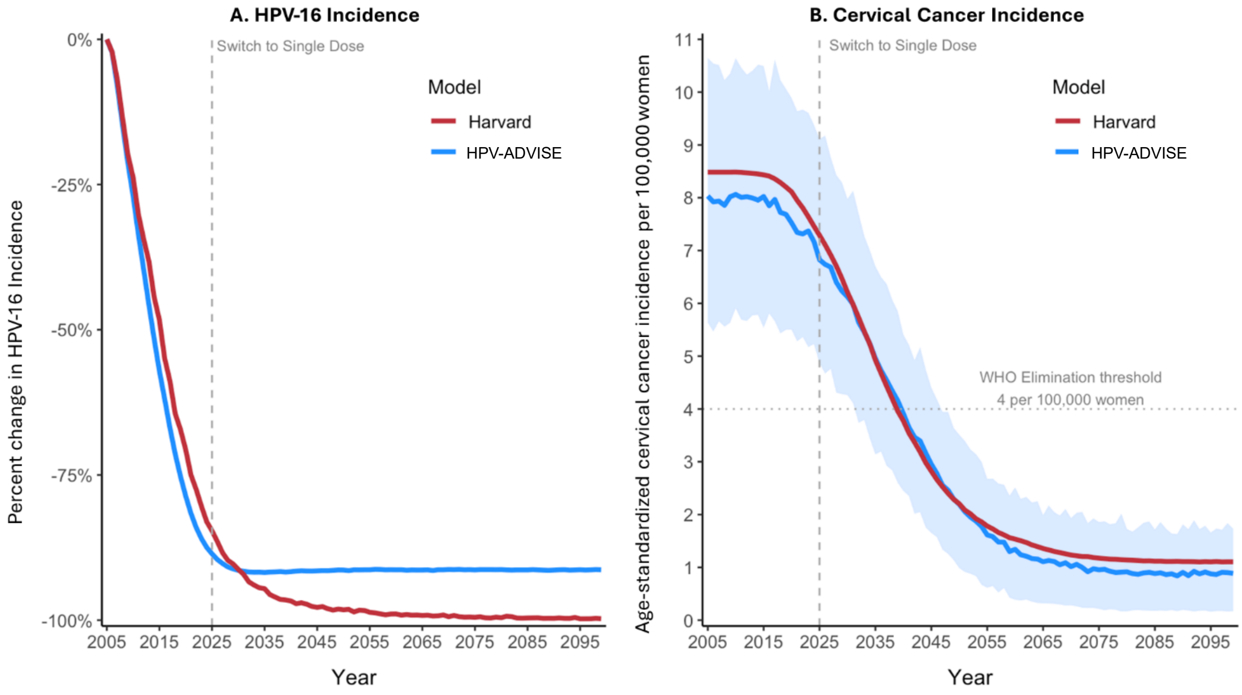

Supplement Figure 9. Projected reductions in age-standardized human papillomavirus (HPV)-16 incidence (**Panel A**) and age-standardized cervical cancer incidence (**Panel B**) from 2005–2099 shown separately for the Harvard and HPV-ADVISE models under lower HPV vaccination coverage for *Scenario 2* (switch to single-dose vaccination in 2025 assuming an average duration of 25 years (5-year standard deviation)). The WHO 2015 female population was used for standardization, consistent with WHO recommendations for cervical cancer elimination projections (5).

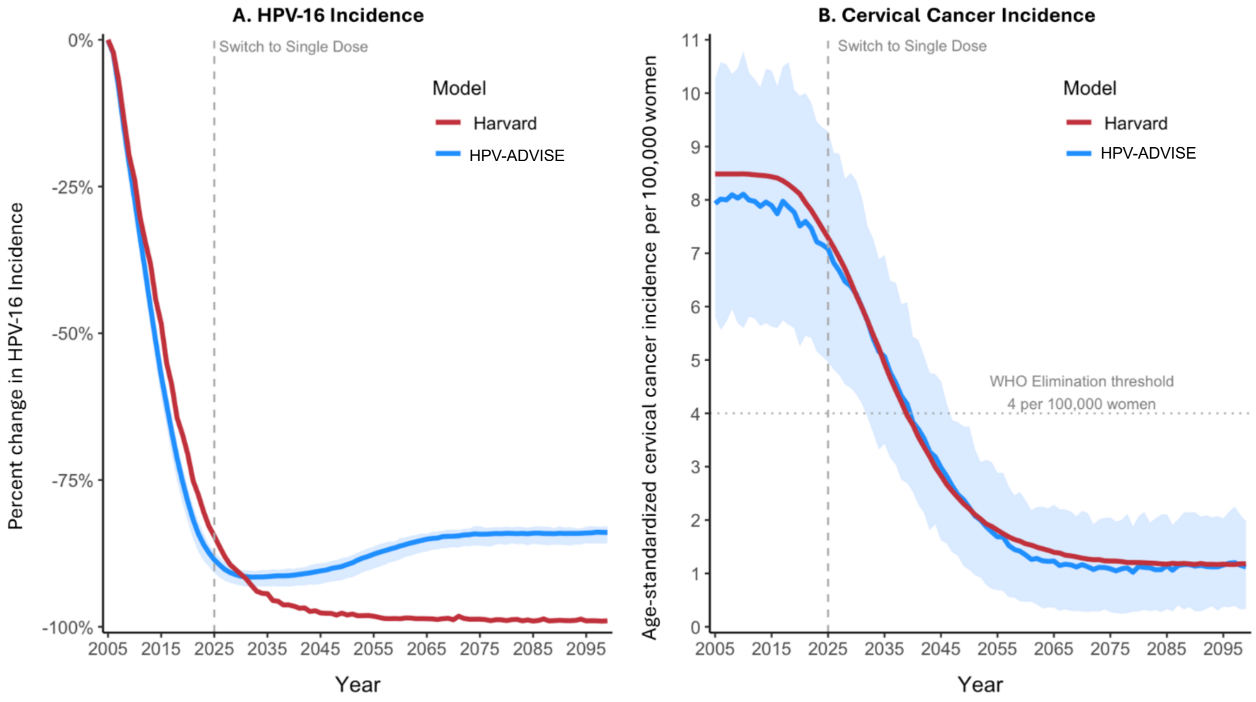

Supplement Figure 10. Projected reductions in age-standardized human papillomavirus (HPV)-16 incidence (**Panel A**) and age-standardized cervical cancer incidence (**Panel B**) from 2005–2099 shown separately for the Harvard and HPV-ADVISE models under lower HPV vaccination coverage for *Scenario 3* (switch to single-dose vaccination in 2025 assuming single-dose vaccine efficacy of 90%). The WHO 2015 female population was used for standardization, consistent with WHO recommendations for cervical cancer elimination projections (5).

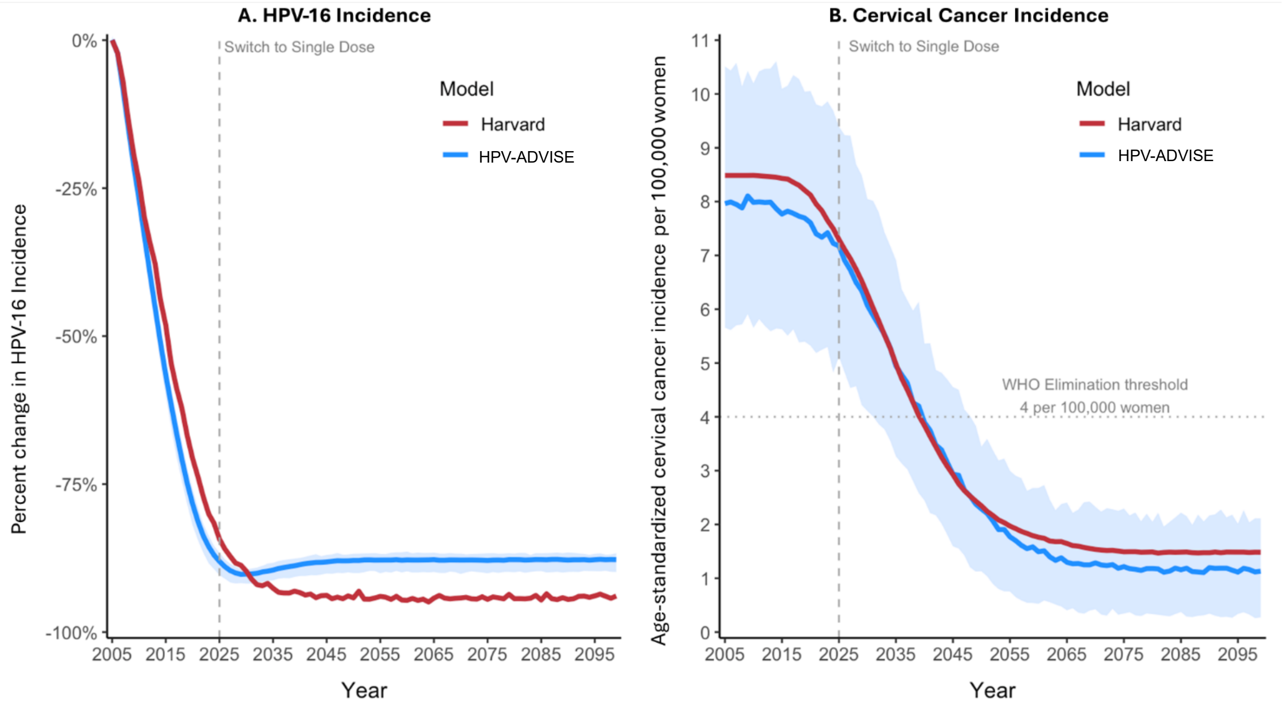

Supplement Figure 11. Projected reductions in age-standardized human papillomavirus (HPV)-16 incidence (**Panel A**) and age-standardized cervical cancer incidence (**Panel B**) from 2005–2099 shown separately for the Harvard and HPV-ADVISE models under lower HPV vaccination coverage for *Scenario 4* (switch to single-dose vaccination in 2025 assuming single-dose vaccine efficacy of 90% and an average duration of 25 years (5-year standard deviation)). The WHO 2015 female population was used for standardization, consistent with WHO recommendations for cervical cancer elimination projections (5).

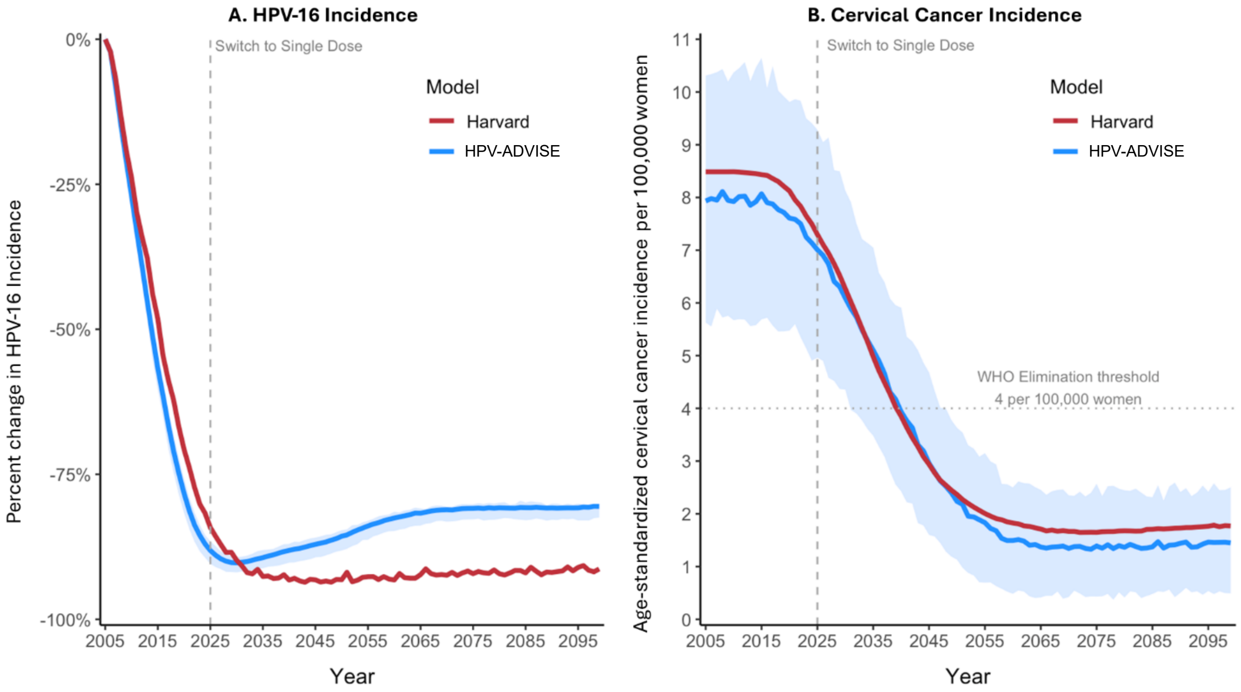

5. Supplement Tables

Supplement Table 1. Model input for the annual probability of receiving 2+ doses of the HPV vaccine for females (shaded area reflects values projected for years 2023+)

Year\Age	>=10	11	12	13	14	15	16	17	18	19	20	21	22	23	24	25	26	27+
**2006**	2·1%	2·6%	4·9%	4·2%	4·5%	6·4%	5·3%	5·3%	4·1%	2·4%	2·8%	2·0%	1·9%	1·6%	2·2%	2·1%	1·9%	0·0%
**2007**	3·7%	15·4%	14·5%	21·5%	21·3%	21·5%	20·7%	12·7%	3·8%	1·9%	2·9%	2·0%	1·8%	1·8%	1·8%	2·5%	1·0%	0·0%
**2008**	4·7%	17·9%	18·1%	18·6%	22·1%	18·1%	16·3%	5·5%	4·5%	1·8%	2·3%	2·1%	1·7%	1·6%	1·9%	2·1%	1·3%	0·0%
**2009**	4·1%	14·8%	12·3%	11·3%	16·0%	13·9%	10·2%	4·4%	4·0%	2·4%	2·2%	1·6%	1·8%	1·6%	1·8%	2·3%	1·1%	0·0%
**2010**	5·0%	17·3%	15·2%	10·0%	9·4%	14·2%	7·8%	2·1%	3·8%	1·9%	3·0%	1·5%	1·4%	1·6%	1·7%	2·1%	1·1%	0·0%
**2011**	6·2%	19·3%	14·7%	18·9%	15·4%	14·8%	10·9%	1·5%	4·2%	1·8%	2·3%	2·1%	1·3%	1·3%	1·8%	2·0%	1·0%	0·0%
**2012**	4·1%	21·8%	18·3%	15·9%	16·2%	11·0%	7·0%	3·3%	3·8%	2·0%	2·2%	1·6%	1·9%	1·2%	1·4%	2·1%	1·0%	0·0%
**2013**	3·8%	20·8%	25·5%	13·2%	17·0%	11·4%	12·2%	2·0%	3·7%	1·8%	2·4%	1·5%	1·4%	1·7%	1·3%	1·6%	1·1%	0·0%
**2014**	6·7%	27·4%	17·8%	15·8%	18·9%	16·3%	10·3%	1·0%	2·4%	1·7%	2·1%	1·7%	1·3%	1·3%	1·9%	1·5%	0·8%	0·0%
**2015**	5·9%	29·0%	24·2%	19·2%	18·4%	11·4%	12·9%	3·2%	2·8%	1·1%	2·1%	1·5%	1·5%	1·2%	1·4%	2·2%	0·8%	0·0%
**2016**	6·6%	32·8%	31·2%	17·5%	14·0%	14·2%	8·6%	7·1%*	3·3%	1·3%	1·4%	1·5%	1·3%	1·4%	1·3%	1·6%	1·1%	0·0%
**2017**	5·1%	41·6%	30·4%	21·4%	25·5%	22·6%	8·6%	2·5%	5·3%*	1·5%	1·6%	0·9%	1·3%	1·2%	1·5%	1·5%	0·8%	0·0%
**2018**	5·6%	37·9%	33·1%	25·1%	16·5%	12·2%	7·9%	3·3%	6·9%	2·5%	1·9%	1·1%	0·8%	1·2%	1·3%	1·7%	0·8%	0·0%
**2019**	9·6%	42·4%	36·7%	16·4%	13·1%	13·3%	19·2%	1·5%	8·0%	3·4%	3·0%	1·3%	1·0%	0·7%	1·3%	1·5%	0·9%	0·0%
**2020**	9·6%	37·6%	27·7%	15·8%	9·4%	6·5%	9·9%	1·5%	16·3%	3·8%	4·2%	2·2%	1·2%	0·8%	0·8%	1·5%	0·8%	0·0%
**2021**	9·6%	37·6%	18·0%	8·9%	8·3%	6·3%	2·2%	2·5%	22·0%	8·6%	4·8%	3·0%	1·9%	1·0%	0·9%	0·9%	0·7%	0·0%
**2022**	9·6%	37·6%	18·0%	0·3%	0·0%	0·4%	4·1%	2·5%	0·0%	12·5%	11·3%	3·5%	2·7%	1·7%	1·1%	1·1%	0·5%	0·0%
**2023+**	9·6%	37·6%	18·0%	0·3%	0·0%	0·4%	4·1%	2·5%	0·0%	11·0%**	8·6%**	3·5%	2·7%	1·7%	1·1%	1·1%	0·5%	0·0%

*Estimated via Excel Solver to fit continuous cumulative birth cohort coverage data; ** Adjusted to ensure cumulative coverage with >=1 dose do not exceed 85% by age 26 years. Estimates rounded to the nearest tenth of a percent.

Supplement Table 2. Model input for the annual probability of receiving 1 dose of the HPV vaccine for females (shaded area reflects values projected for years 2023+)

Year\Age	>=10	11	12	13	14	15	16	17	18	19	20	21	22	23	24	25	26	27+
**2006**	0·0%	0·3%	0·0%	0·3%	0·1%	1·7%	1·2%	1·2%	0·8%	0·6%	0·8%	0·6%	0·5%	0·8%	0·5%	0·9%	1·1%	0·0%
**2007**	0·0%	0·3%	1·0%	0·5%	0·8%	1·5%	4·1%	2·9%	1·2%	0·4%	0·7%	0·6%	0·5%	0·4%	0·8%	0·6%	0·5%	0·0%
**2008**	0·0%	0·7%	0·9%	1·2%	1·9%	3·8%	4·1%	5·2%	13·8%	0·6%	0·5%	0·5%	0·5%	0·5%	0·5%	1·0%	0·3%	0·0%
**2009**	0·1%	1·9%	1·1%	1·5%	1·1%	3·6%	3·2%	4·8%	1·8%	0·7%	0·7%	0·3%	0·4%	0·5%	0·5%	0·5%	0·5%	0·0%
**2010**	0·1%	0·4%	1·1%	1·5%	0·6%	1·8%	5·2%	4·5%	1·6%	0·9%	0·8%	0·5%	0·3%	0·4%	0·5%	0·6%	0·3%	0·0%
**2011**	0·0%	0·9%	0·7%	1·6%	1·9%	2·2%	4·0%	4·6%	1·9%	0·8%	1·1%	0·6%	0·4%	0·2%	0·4%	0·6%	0·3%	0·0%
**2012**	0·0%	1·4%	1·9%	2·2%	1·6%	3·6%	1·9%	4·3%	0·8%	0·9%	0·9%	0·8%	0·5%	0·4%	0·3%	0·5%	0·3%	0·0%
**2013**	0·4%	1·6%	1·6%	1·4%	1·3%	3·8%	5·3%	2·7%	1·6%	0·4%	1·1%	0·7%	0·7%	0·5%	0·4%	0·3%	0·3%	0·0%
**2014**	0·2%	0·8%	0·5%	1·0%	0·9%	2·9%	4·1%	3·3%	0·7%	0·7%	0·5%	0·8%	0·6%	0·6%	0·5%	0·5%	0·2%	0·0%
**2015**	0·1%	0·6%	1·5%	1·7%	1·3%	2·3%	5·4%	3·4%	1·3%	0·3%	0·9%	0·3%	0·7%	0·5%	0·7%	0·6%	0·2%	0·0%
**2016**	0·0%	1·2%	2·0%	2·8%	2·0%	0·9%	4·9%	0·4%*	2·0%	0·6%	0·4%	0·6%	0·3%	0·6%	0·6%	0·8%	0·3%	0·0%
**2017**	0·2%	1·7%	2·0%	1·0%	2·7%	1·3%	5·0%	6·7%	0·4%*	0·9%	0·7%	0·3%	0·6%	0·3%	0·7%	0·7%	0·4%	0·0%
**2018**	0·2%	2·3%	2·7%	0·8%	4·3%	6·8%	4·5%	4·8%	1·5%	0·2%	1·2%	0·5%	0·2%	0·5%	0·3%	0·8%	0·3%	0·0%
**2019**	0·4%	3·7%	7·0%	4·6%	2·2%	1·1%	6·1%	5·3%	0·0%	0·7%	0·2%	0·8%	0·4%	0·2%	0·5%	0·3%	0·4%	0·0%
**2020**	0·4%	7·7%	7·5%	7·9%	2·8%	2·3%	6·8%	5·4%	0·0%	0·0%	0·9%	0·2%	0·7%	0·4%	0·2%	0·6%	0·2%	0·0%
**2021**	0·4%	7·7%	20·0%	9·5%	5·4%	4·9%	2·4%	5·0%	0·0%	0·0%	0·0%	0·6%	0·2%	0·6%	0·4%	0·3%	0·3%	0·0%
**2022**	0·4%	7·7%	20·0%	10·2%	12·6%	2·3%	6·7%	5·6%	0·0%	0·0%	0·0%	0·0%	0·6%	0·1%	0·7%	0·5%	0·1%	0·0%
**2023+**	0·4%	7·7%	20·0%	9·0%**	7·5%**	2·5%**	5·5%**	4·5%**	0·0%	0·0%	0·0%	0·0%	0·4%	0·1%	0·4%	0·5%	0·1%	0·0%

*Estimated via Excel Solver to fit continuous positive increase in cumulative coverage by birth cohort; ** Adjusted to ensure cumulative coverage with >=1 dose do not exceed 85% by age 26 years. Estimates rounded to the nearest tenth of a percent.

Supplement Table 3. Model input for the annual probability of receiving 2+ doses of the HPV vaccine for males (shaded area reflects values projected for years 2023+)

Year\Age	>=10	11	12	13	14	15	16	17	18	19	20	21	22	23	24	25	26	27+
**2006**	0·0%	0.0%	0.0%	0.0%	0.0%	0.0%	0.0%	0.0%	0.0%	0.0%	0.0%	0.0%	0.0%	0.0%	0.0%	0.0%	0.0%	0.0%
**2007**	0·0%	0.0%	0.0%	0.0%	0.0%	0.0%	0.0%	0.0%	0.0%	0.0%	0.0%	0.0%	0.0%	0.0%	0.0%	0.0%	0.0%	0.0%
**2008**	0·0%	0.0%	0.0%	0.0%	0.0%	0.0%	0.0%	0.0%	0.0%	0.0%	0.0%	0.0%	0.0%	0.0%	0.0%	0.0%	0.0%	0.0%
**2009**	0·0%	0.0%	0.0%	0.0%	0.0%	0.0%	0.0%	0.0%	0.0%	0.0%	0.0%	0.0%	0.0%	0.0%	0.0%	0.0%	0.0%	0.0%
**2010**	0·0%	0.0%	0.0%	0.0%	0.0%	0.0%	0.0%	0.0%	0.0%	0.0%	0.0%	0.0%	0.0%	0.0%	0.0%	0.0%	0.0%	0.0%
**2011**	1·4%	7.0%	6.8%	7.0%	9.1%	6.5%	5.4%	1.0%	1.2%	0.4%	1.1%	0.0%*	0.0%*	0.0%*	0.0%*	0.0%*	0.0%*	0.0%*
**2012**	2·6%	13.0%	11.3%	10.3%	11.2%	11.8%	9.8%	2.9%	1.4%	0.5%	0.4%	0.0%*	0.0%*	0.0%*	0.0%*	0.0%*	0.0%*	0.0%*
**2013**	2·2%	20.2%	15.8%	13.0%	14.6%	12.0%	9.4%	1.8%	1.8%	0.5%	0.4%	0.0%*	0.0%*	0.0%*	0.0%*	0.0%*	0.0%*	0.0%*
**2014**	2·0%	22.5%	14.4%	13.8%	16.4%	12.3%	12.1%	2.7%	1.8%	0.7%	0.4%	0.0%*	0.0%*	0.0%*	0.0%*	0.0%*	0.0%*	0.0%*
**2015**	4·7%	26.0%	18.6%	12.5%	12.8%	12.8%	8.9%	2.1%	2.5%	0.7%	0.6%	0.0%*	0.0%*	0.0%*	0.0%*	0.0%*	0.0%*	0.0%*
**2016**	3·9%	29.9%	26.7%	21.5%	16.0%	11.5%	11.9%	2.2%	1.2%	1.0%	0.6%	0.0%*	0.0%*	0.0%*	0.0%*	0.0%*	0.0%*	0.0%*
**2017**	4·6%	36.2%	27.3%	22.9%	16.2%	12.7%	9.6%	2.2%	1.8%	0.5%	0.8%	0.0%*	0.0%*	0.0%*	0.0%*	0.0%*	0.0%*	0.0%*
**2018**	6·6%	35.9%	27.4%	15.3%	17.2%	9.0%	12.1%	1.9%	0.9%	0.6%	0.4%	0.0%*	0.0%*	0.0%*	0.0%*	0.0%*	0.0%*	0.0%*
**2019**	5·1%	40.0%	27.6%	21.5%	19.6%	9.3%	5.0%	2.3%	4.5%	0.3%	0.5%	0.0%	0.0%	0.0%	0.0%	0.0%	0.0%	0.0%
**2020**	5·1%	35.9%	24.8%	17.4%	9.3%	9.9%	7.1%	1.1%	15.9%	1.8%	0.3%	0.3%	0.2%	0.3%	0.3%	0.3%	0.2%	0.0%
**2021**	5·1%	35.9%	16.0%	7.0%	7.7%	9.1%	2.9%	3.2%	3.6%	7.1%	1.5%	0.2%	0.3%	0.1%	0.4%	0.3%	0.2%	0.0%
**2022**	5·1%	35.9%	16.0%	0.4%	0.2%	1.1%	2.3%	1.6%	3.7%	1.4%	6.4%	0.8%	0.2%	0.2%	0.2%	0.4%	0.2%	0.0%
**2023+**	5·1%	35.9%	16.0%	0.4%	0.2%	1.1%	2.3%	1.6%	3.7%	1.4%	6.4%	0.8%	0.2%	0.2%	0.2%	0.4%	0.2%	0.0%

*Assumed zero to reflect US ACIP policy for males >20 years prior to 2019. Estimates rounded to the nearest tenth of a percent.

Supplement Table 4. Model input for the annual probability of receiving 1 dose of the HPV vaccine for males (shaded area reflects values projected for years 2023+)

**Year\Age**	**>=10**	**11**	**12**	**13**	**14**	**15**	**16**	**17**	**18**	**19**	**20**	**21**	**22**	**23**	**24**	**25**	**26**	**27+**
**2006**	0·0%	0·0%	0·0%	0·0%	0·0%	0·0%	0·0%	0·0%	0·0%	0·0%	0·0%	0·0%	0·0%	0·0%	0·0%	0·0%	0·0%	0·0%
**2007**	0·0%	0·0%	0·0%	0·0%	0·0%	0·0%	0·0%	0·0%	0·0%	0·0%	0·0%	0·0%	0·0%	0·0%	0·0%	0·0%	0·0%	0·0%
**2008**	0·0%	0·0%	0·0%	0·0%	0·0%	0·0%	0·0%	0·0%	0·0%	0·0%	0·0%	0·0%	0·0%	0·0%	0·0%	0·0%	0·0%	0·0%
**2009**	0·0%	0·0%	0·0%	0·0%	0·0%	0·0%	0·0%	0·0%	0·0%	0·0%	0·0%	0·0%	0·0%	0·0%	0·0%	0·0%	0·0%	0·0%
**2010**	0·0%	0·0%	0·0%	0·0%	0·0%	0·0%	0·0%	0·0%	0·0%	0·0%	0·0%	0·0%	0·0%	0·0%	0·0%	0·0%	0·0%	0·0%
**2011**	0·0%	0·2%	2·2%	0·5%	0·5%	1·4%	3·0%	2·3%	0·6%	0·4%	0·5%	0·0%*	0·0%*	0·0%*	0·0%*	0·0%*	0·0%*	0·0%*
**2012**	0·0%	1·3%	1·4%	0·9%	1·5%	1·7%	3·8%	4·3%	0·8%	0·2%	0·4%	0·0%*	0·0%*	0·0%*	0·0%*	0·0%*	0·0%*	0·0%*
**2013**	0·1%	0·9%	1·7%	1·0%	1·1%	2·2%	5·1%	4·2%	0·7%	0·3%	0·2%	0·0%*	0·0%*	0·0%*	0·0%*	0·0%*	0·0%*	0·0%*
**2014**	0·0%	0·6%	1·2%	0·8%	1·6%	1·9%	5·5%	3·9%	1·1%	0·3%	0·3%	0·0%*	0·0%*	0·0%*	0·0%*	0·0%*	0·0%*	0·0%*
**2015**	0·0%	1·1%	1·2%	1·8%	2·8%	2·9%	8·9%	3·3%	1·4%	0·4%	0·2%	0·0%*	0·0%*	0·0%*	0·0%*	0·0%*	0·0%*	0·0%*
**2016**	0·0%	1·5%	2·8%	2·4%	1·7%	1·1%	6·3%	4·4%	1·4%	0·5%	0·4%	0·0%*	0·0%*	0·0%*	0·0%*	0·0%*	0·0%*	0·0%*
**2017**	0·0%	1·8%	1·5%	1·1%	3·0%	7·1%	4·3%	4·1%	1·4%	0·5%	0·5%	0·0%*	0·0%*	0·0%*	0·0%*	0·0%*	0·0%*	0·0%*
**2018**	0·0%	4·8%	3·1%	1·6%	2·8%	5·2%	4·7%	4·0%	0·7%	0·3%	0·5%	0·0%*	0·0%*	0·0%*	0·0%*	0·0%*	0·0%*	0·0%*
**2019**	0·2%	2·6%	5·6%	3·6%	1·6%	9·0%	8·5%	4·9%	0·0%	0·2%	0·3%	0·0%	0·0%	0·0%	0·0%	0·0%	0·0%	0·0%
**2020**	0·2%	6·0%	13·6%	4·3%	3·2%	1·5%	3·6%	7·3%	0·0%	0·0%	0·2%	0·2%	0·3%	0·2%	0·2%	0·1%	0·1%	0·0%
**2021**	0·2%	6·0%	13·9%	9·7%	14·4%	5·5%	2·4%	1·2%	0·0%	0·0%	0·0%	0·1%	0·2%	0·2%	0·2%	0·2%	0·1%	0·0%
**2022**	0·2%	6·0%	13·9%	8·7%	9·3%	1·7%	3·0%	4·0%	0·0%	0·0%	0·0%	0·0%	0·1%	0·1%	0·2%	0·2%	0·1%	0·0%
**2023+**	0·2%	6·0%	13·9%	8·7%	9·3%	1·7%	3·0%	4·0%	0·0%	0·0%	0·0%	0·0%	0·1%	0·1%	0·2%	0·2%	0·1%	0·0%

*Assumed zero to reflect US ACIP policy for males >20 years prior to 2019. Estimates rounded to the nearest tenth of a percent.

Supplement Table 5. Model output of cumulative coverage of the human papillomavirus (HPV) vaccine by age 17 and 26 years by birth cohort, number of doses, and sex (shaded area reflects accumulated coverage for future birth cohorts)

	Cumulative by age 17				Cumulative by age 26			
	Females		Males		Females		Males	
Birth cohort	≥1 Dose	≥2 Doses	≥1 Dose	≥2 Doses	≥1 Dose	≥2 Doses	≥1 Dose	≥2 Doses
1980	0·0%	0·0%	0·0%	0·0%	3·0%	1·9%	0·0%	0·0%
1981	0·0%	0·0%	0·0%	0·0%	4·5%	3·1%	0·0%	0·0%
1982	0·0%	0·0%	0·0%	0·0%	7·2%	5·8%	0·0%	0·0%
1983	0·0%	0·0%	0·0%	0·0%	9·4%	6·5%	0·0%	0·0%
1984	0·0%	0·0%	0·0%	0·0%	10·7%	8·7%	0·0%	0·0%
1985	0·0%	0·0%	0·0%	0·0%	12·5%	9·8%	0·0%	0·0%
1986	0·0%	0·0%	0·0%	0·0%	15·6%	12·0%	0·0%	0·0%
1987	0·0%	0·0%	0·0%	0·0%	18·0%	14·5%	0·0%	0·0%
1988	0·0%	0·0%	0·0%	0·0%	18·1%	15·2%	0·0%	0·0%
1989	6·5%	5·3%	0·0%	0·0%	23·8%	18·5%	0·0%	0·0%
1990	21·0%	17·1%	0·0%	0·0%	47·8%	30·3%	0·0%	0·0%
1991	38·2%	29·2%	0·0%	0·0%	51·6%	38·3%	1·5%	1·1%
1992	47·0%	39·6%	0·0%	0·0%	57·5%	47·0%	1·6%	0·8%
1993	53·0%	45·0%	0·0%	0·0%	63·7%	52·3%	3·1%	2·1%
1994	62·1%	53·8%	3·3%	1·0%	68·6%	59·1%	7·2%	3·4%
1995	64·0%	56·5%	15·0%	8·1%	71·0%	61·4%	19·3%	11·1%
1996	62·2%	56·8%	25·3%	17·0%	66·7%	60·2%	29·9%	19·8%
1997	66·5%	57·6%	37·6%	29·0%	71·5%	61·1%	42·9%	32·4%
1998	70·6%	61·7%	46·0%	36·6%	76·1%	65·2%	49·5%	38·3%
1999	72·7%	66·2%	55·0%	42·6%	78·1%	71·0%	58·3%	44·5%
2000	72·0%	63·4%	63·1%	53·4%	79·4%	69·5%	64·9%	54·4%
2001	76·3%	69·8%	64·4%	55·6%	82·3%	75·5%	68·0%	59·0%
2002	74·9%	67·6%	71·6%	61·3%	84·9%	77·4%	79·8%	69·3%
2003	83·5%	74·4%	74·8%	63·7%	90·9%	81·7%	78·2%	67·0%
2004	82·3%	75·5%	78·6%	70·0%	87·2%	80·2%	81·5%	72·7%
2005	79·0%	73·0%	75·9%	70·0%	84·9%	78·7%	79·1%	73·1%
2006	82·6%	73·9%	79·3%	70·1%	87·5%	78·5%	82·1%	72·8%
2007	82·8%	70·2%	79·9%	64·4%	87·6%	74·8%	82·6%	67·0%
2008	80·8%	64·1%	78·4%	60·9%	86·2%	69·2%	81·3%	63·6%
2009	79·4%	54·1%	72·5%	49·7%	85·2%	59·6%	76·2%	53·2%
2010+	79·2%*	54·1%*	72·5%	49·7%	85·0%*	59·7%*	76·2%	53·2%

* Annual probability of being vaccinated from 2023 onward adjusted to ensure cumulative coverage with >=1 dose do not exceed 85% by age 26 years. Estimates rounded to the nearest tenth of a percent.

Supplement Table 6. Summary of modeled scenarios

Scenario	Efficacy single dose vaccination (%)	Duration of protection for Single dose Vaccination	Schedule	Year switch to single dose	Single-dose schedule target population
1*	98	Lifelong	2 doses (or single dose with equivalent protection)	N/A	N/A
2	98	Mean 25 years (SD 5)	Single dose	2025	Females and males: 9–20 years
3	90	Lifelong	Single dose	2025	Females and males: 9–20 years
4	90	Mean 25 years (SD 5)	Single dose	2025	Females and males: 9–20 years

*Continued use of 2-dose program, or single-dose with efficacy/duration equivalent to 2-dose

6. Supplement References

1. Centers for Disease Control and Prevention. NHANES - National Health and Nutrition Examination Survey. National Center for Health Statistics. 2002 to 2016. Accessed January 15, 2023. https://www.cdc.gov/nchs/nhanes/index.htm [Internet].
2. Brisson M, Laprise J-F, Drolet M, Chamberland É, Bénard É, Burger EA, et al. Population-level impact of switching to 1-dose human papillomavirus vaccination in high-income countries: examining uncertainties using mathematical modeling. JNCI Monographs. 2024;2024(67):387-99.
3. Drolet M, Laprise J-F, Chamberland É, Sauvageau C, Wilson S, Lim GH, et al. Switching from a 2-dose to a 1-dose program of gender-neutral routine vaccination against human papillomavirus in Canada: a mathematical modelling analysis. Canadian Medical Association Journal. 2024;196(33):E1136-E43.
4. Daniels V, Saxena K, Patterson-Lomba O, Gomez-Lievano A, Saah A, Luxembourg A, et al. Modeling the health and economic implications of adopting a 1-dose 9-valent human papillomavirus vaccination regimen in a high-income country setting: An analysis in the United Kingdom. Vaccine. 2022;40(14):2173-83.
5. United Nations Population Division. World Population Prospects: The 2017 Revision. [Online] Accessed 18 December 2017. Available at: https://esa.un.org/unpd/wpp/.
