# Supplementary material for: Single-dose HPV vaccination in the United States — a multi-modeling analysis
Source: Lancet Reg Health Am. 2026 Jan 10;55:101361. doi: 10.1016/j.lana.2025.101361 (PMC12825051; doi:10.1016/j.lana.2025.101361)
Supplement: HPV-FRAME checklist [file mmc2.pdf]

S1. HPV-FRAME reporting standard checklist

The HPV-FRAME checklist is shown below for the core reporting standard and reporting standard for models of HPV vaccination in adolescents and adults [1].

**Inputs**	**Reported by age? (Y/N)**		**Report by sex (F/M/Both)?**		**Comments**
	Harvard	HPV-ADVISE	Harvard	HPV-ADVISE	
Target population for intervention	Y	Y	Y	Y	Described in methods section of the main manuscript
Sexual behavior	Y	Y	Y	Y	Harvard and HPV-ADVISE provide detailed information in their technical appendices
Cohort examined for evaluation/ time horizon	Y (multiple cohorts)	Y (multiple cohorts)	Y (multiple cohorts)	Y (multiple cohorts)	Results incorporate multiple birth cohorts as described in the methods section of the main manuscript. The time horizon is also described here.
Quality of life assumptions	N/A	N/A	N/A	N/A	N/A for health impact analysis
Calibration	Y	Y	Y	Y	Calibration against genital HPV prevalence by age and genotype provided for both Harvard and HPV-ADVISE in technical appendices

Validation (where possible)	Y	Y	N/A	N/A	Using observed HPV vaccination coverage and uptake by sex, age and period, both models compared model-predicted HPV-16 prevalence against available empirical data (Figure 2)
Costs	N/A	N/A	N/A	N/A	N/A for health impact analysis
Vaccine coverage	Y	Y	Y	Y	Described in the main manuscript and in supplementary appendix
Vaccine uptake	Y	Y	Y	Y	Described in the main manuscript and in supplementary appendix
Vaccine efficacy	Y	Y	N	N	Assumptions described in '*Vaccination scenarios: single-dose efficacy and durability'* section
Vaccine cross-protection	N/A	N/A	N/A	N/A	Efficacy is assumed to apply against vaccine-included types only, as described in the main manuscript.
Duration vaccine protection and waning	Y	Y	N	N	Assumptions described in '*Vaccination scenarios: single-dose efficacy and durability'* section
Vaccine and delivery costs	N/A	N/A	N/A	N/A	N/A for health impact analysis

Pre-vaccination disease burden (including population attributable fractions for HPV)	Y	Y	Y	Y	Model-predicted pre-vaccination genital HPV prevalence by age and genotype is described in model technical appendices and compared to real-world observed data
Duration of natural immunity	Y	Y	Y	Y	Lifelong natural immunity (Table 1)
Natural history parameters, specifically for older individuals	Y	Y	Y	Y	Harvard and HPV-ADVISE provide detailed information in their technical appendices about HPV infection natural history<br><br>The Harvard model has reported cervical cancer natural history parameters by age, time since infection and HPV type in prior analyses (cited in current analysis). See HPV-ADVISE technical appendix for cervical cancer natural history parameters<br><br>Re-activation not modelled by either group; infections in older women are assumed to be new infections
Screening impact for vaccinated individuals	N/A	N/A	N/A	N/A	Screening not assumed to change, and vaccination coverage independent of screening adherence
**Outputs**	**Reported by age? (Y/N)**		**Report by sex (F/M/Both)?**		**Comments**
Cancer incidence, mortality, life years, QALYs/DALYs (as appropriate)	Y	Y	N	N	Both models report on cervical cancer incidence in current analysis. Life years, QALYs, DALYs outside the scope of the current analysis

HPV prevalence, pre-intervention	Y	Y	Y	Y	Both models (technical appendices) report on female and male genital HPV prevalence pre-intervention as a calibration target against observed data, by age and HPV genotype.
CIN2/3 detected	N	N	N	N	CIN2/3 was not reported as a separate output
Sensitivity analysis on key inputs	Y	Y	N/A	N/A	Scenarios reflected variation in efficacy and duration of single-dose HPV vaccination. Sensitivity analysis on vaccination coverage was reported
Incremental cost-effectiveness ratios and costs saved	N/A	N/A	N/A	N/A	N/A for health impact analysis
Absolute reductions in HPV infections, and/or warts, post-vaccination	N	N	N	N	Not reported explicitly, although both models capture reductions in infection and disease
Absolute reductions in invasive cancer (cervical and other HPV cancers, as relevant) post-vaccination	N	N	N	N	Absolute reductions in cervical cancer incidence post-vaccination was a main outcome of the analysis.

Reference:

1. Canfell K, Kim JJ, Kulasingam S, Berkhof J, Barnabas R, Bogaards JA, et al. HPV-FRAME: A consensus statement and quality framework for modelled evaluations of HPV-related cancer control. *Papillomavirus Res*. 2019;8:100184. doi:10.1016/j.pvr.2019.100184.
